# Synthesis, Characterization, and NH_3_-SCR Catalytic Performance of Fe-Modified MCM-36 Intercalated with Various Pillars

**DOI:** 10.3390/molecules28134960

**Published:** 2023-06-24

**Authors:** Agnieszka Szymaszek-Wawryca, Urbano Díaz, Bogdan Samojeden, Monika Motak

**Affiliations:** 1 Faculty of Energy and Fuels, AGH University of Science and Technology, Al. Mickiewicza 30, 30-059 Kraków, Poland; bsamo1@agh.edu.pl (B.S.); motakm@agh.edu.pl (M.M.); 2 Instituto de Tecnología Química, UPV-CSIC, Universidad Politécnica de Valencia, Avenida de los Naranjos, s/n., 46022 Valencia, Spain; udiaz@itq.upv.es

**Keywords:** MWW zeolites, MCM-36, iron, alumina, intercalation, DeNOx

## Abstract

Two series of MCM-36 zeolites intercalated with various pillars and modified with iron were synthesized, analyzed with respect to their physicochemical properties, and tested as catalysts for the NH_3_-SCR process. It was found that the characteristic MWW morphology of MCM-36 can be obtained successfully using silica, alumina, and iron oxide as pillars. Additionally, one-pot synthesis of the material with iron resulted in the incorporation of monomeric Fe^3+^ species into the framework positions. The results of catalytic tests revealed that the one-pot synthesized sample intercalated with silica and alumina was the most efficient catalyst of NO reduction, exhibiting ca. 100% activity at 250 °C. The outstanding performance of the material was attributed to the abundance of Lewis acid sites and the beneficial influence of alumina on the distribution of iron species in the zeolite. In contrast, the active centers originating from the Fe_2_O_3_ pillars improved the NO conversion in the high-temperature range. Nevertheless, the aggregated particles of the metal oxide limited the access of the reacting molecules to the inner structure of the catalyst, which affected the overall activity and promoted the formation of N_2_O above 300 °C.

## 1. Introduction

Layered zeolites belonging to the MWW (Mobile Twenty-Two) family are an important group of materials which exhibit very attractive structural properties. The discovery of the layered precursor, MCM-22P, has been widely accepted as the starting point in the evolution of the MWW zeolites. Their topology consists of two independent pore systems in the 2.5 nm-thick layer, formed by a 10-member ring (MR), 2D sinusoidal channels, and by large supercages (0.71 nm × 0.71 nm × 1.82 nm). The distinctive feature of these zeolites is the high concentrations of their 12-MR external pockets of an approximate depth of 0.71 nm [1]. Due to their unique physicochemical characterization, such as a well-developed pore structure, high hydrothermal stability, and mild acid performance, these materials have found many practical applications in the industry [2,3].

One of the biggest commercial implementations of the MWW zeolites was the introduction of MCM-22 by Exxon-Mobil Company in the production of cumene by liquid-phase alkylation in 1990 [4]. MCM-22 is typically obtained upon the calcination of MCM-22P, which results in the formation of another 10-MR window, and the connection of the adjacent MWW layers by the condensation of the Si-OH groups. As a result, MCM-36 12-MRs and 1.82 nm high MWW cages are formed simultaneously with the 3D zeolitic structure [5]. Furthermore, in 1993, Bennett et al. [6] prepared the material named MCM-49, which exhibits a framework topology identical to that of MCM-22. However, in order to prepare MCM-49, it is required to use the molar ratio of the organic template (hexamethyleneimine, HMI) to inorganic alkali cations lower than 2.0. Additionally, the specific surface area of MCM-49 is slightly higher compared to MCM-22 [7]. Another interesting representative of these MWW zeolites is MCM-56, which is an intermediate in the synthesis of MCM-49. According to the literature, the optimal time window for the formation of MCM-56 is 2–3 h, and thus, it must be isolated before MCM-49 is fully crystalized. In general, the main feature differing these two zeolites is the unit cell crystal thickness, which can be easily identified from the XRD patterns [7]. Another significant modification of MCM-22P, patented by Corma et al. [8], involves its swelling with centyltrimethylammonium bromide (CTABr), followed by delamination to yield ITQ-2. The structure of the material can be formed using single 2.5 nm-thick MWW layers, organized in a characteristic “house of cards” arrangement [9].

Apart from the above-mentioned, one of the most interesting members of the MWW zeolites is MCM-36, the pillared zeolite. Due to its outstanding structural parameters, the material found its application as a catalyst for many important chemical processes, based on both organic [10,11,12] and inorganic [5,9,13] reactions. Typically, the synthesis of MCM-36 starts from the swelling of MCM-22P with CTABr and further intercalation with the precursor of silica, tetraethoxysilane (TEOS), which is hydrolyzed in the final preparation step [14]. The swelling treatment is necessary to expand the interlayer distance, while the introduction of pillars stabilizes the swollen structure [15]. Separation of the individual MWW layers after calcination of the intercalated material forms slit-like, characteristic mesopores in the interlayer space. Moreover, aside from mesopores, MCM-36 has a microporous texture inside these MWW layers [7]. Thus, the morphology of the material is very similar to that of natural pillared clays [16,17].

Importantly, in contrast to other mesoporous materials, including MCM-41, the mesopores of MCM-36 are characterized with an irregular size. Additionally, the distance between the adjacent MWW layers has been strongly correlated with the applied swelling conditions. Thus, the physicochemical features of MCM-36 can be tailored through various strategies, such as facilitating the introduction of various oxides in a form of pillars other than silica. The series of studies reported by Kornatowski et al. [18,19,20] suggested that apart from SiO_2_, also Al_2_O_3_, BaO, MgO, and their mixtures can be incorporated into the interlayer space of MCM-36. According to the authors, the results of intercalation with alumina strongly depends on the aging time of the pillaring solution, which should be sufficient to form Keggin ions. Moreover, the zeolite with Al_2_O_3_ was characterized with a lower specific surface area compared to that with silica. It contrast, the co-existence of Al_2_O_3_ and MgO enhanced the formation of a mesoporous texture and improved the incorporation of alumina into the zeolite. The continuation of the research [20] concerned the investigation of the acid–base properties of MCM-36 pillared with binary oxides, e.g., BaO-, MgO–Al_2_O_3_, or SiO_2_–Al_2_O_3_, respectively. It was found that the presence of Al_2_O_3_ as pillars enhanced the Lewis acidity, while BaO and MgO increased the strength of the Brönsted sites of the interlayer silica–alumina clusters. Additionally, barium and magnesium oxides, as well as Al-OH groups on the spinel-type oxide clusters, were also uncovered as the source of the basic centers. Last, but not least, MCM-36 pillared with Al_2_O_3_, BaO, MgO, SiO_2_, or their mixtures were examined for their adsorption features [18]. It was demonstrated that the introduction of silica promoted the expansion of the MWW layers, while the presence of other metal oxides created new adsorption centers. In addition, the co-existence of various pillars was deemed to be beneficial for the stability of the material and improved the sorption properties, especially in the case of smaller mesopores. Moreover, the mesopores formed in the presence of silica showed a broader size distribution and a higher volume. Interestingly, the authors found that the incorporation of three composite pillars yields MCM-36 with the highest sorption capacity. On the other hand, it was also suggested that pillaring can be unfavorable for microporosity since pores with a radius below 2 nm can be almost completely clogged upon intercalation. The alternative and interesting attempt reported in the literature was the introduction of Si/Ti mixed oxides as pillars of MCM-36 [13,21]. According to Fang et al. [22], who assessed Ti-MCM-36 as an epoxidation catalyst, the intercalation of MCM-36 with titania enabled them to obtain material with acid sites originating from the framework Al species and oxidation centers delivered by the Ti species. Furthermore, the authors found that the simultaneous introduction of SiO_2_ and TiO_2_ improved the specific surface area and the mesopore volume of MCM-36 [23]. The silica- and silica-titania-intercalated MCM-36 modified with iron were also prepared by the group of Chmielarz and co-workers [13] and was assessed as a catalyst for the selective catalytic reduction of nitrogen oxides (NH_3_-SCR). The reported results indicated that the highest NO conversion was obtained for the samples which were the most abundant in the TiO_2_ pillars.

According to the above-mentioned, MCM-36 was assessed in several environmentally important industrial processes, for example in the elimination of nitrogen oxides (NO_x_) via NH_3_-SCR. The significance of this technology is related to the toxicity of nitrogen oxides and/or their contribution to ozone layer depletion, acid rain, and photochemical smog formation. Despite the high efficiency of NH_3_-SCR, there are some important problems related to the commercial vanadium-based catalyst, which is widely used on an industrial scale [24,25]. In fact, as postulated by Liang and co-workers in the series of papers [26,27,28], NO can be reduced electrochemically with the simultaneous generation of ammonia. However, the best solution for the existing NH_3_-SCR installations would be to replace the commercial catalyst with another, more ecologically friendly material. Several studies have found modified MCM-36 to be a promising substitutive catalyst for NH_3_-SCR. Rutkowska et al. [5] reported that MCM-36 modified with copper by ion-exchange exhibited almost a 100% NO conversion within 230–530 °C. Moreover, in our previous study [9], we compared the catalytic performances of one-pot synthesized Fe-MCM-22, Fe-MCM-36, and Fe-ITQ-2. Based on our research, we concluded that Fe-MCM-36 showed the superior potential as the catalyst for NO reduction, especially due to the presence of SiO_2_ pillars, which contributed to the formation of new acid centers and towards the well-developed texture of the catalyst. Additionally, we have since confirmed that one-pot synthesis is a very beneficial method to obtain an advantageous distribution of the active phase, which facilitates low-temperature activity in NH_3_-SCR. The positive effect of the introduction of iron into the synthesis pot of MWW zeolites on the catalytic performance was also confirmed in our study on Fe-MCM-22 [29].

As already mentioned, MCM-36 exhibits an analogous morphology to natural clays pillared with various oxides. Considering practical applications, layered zeolites are much more advantageous compared to the natural aluminosilicates, due to their higher flexibility in tailoring of their texture, superior ion-exchange properties, and surface acidity. However, morphological similarities between these two groups of materials led to the conclusion that MCM-36 can be intercalated with the same pillars as the natural clays, e.g., Fe, Ti, Zr, and Al [30,31,32,33]. Taking into account the exceptional NH_3_-SCR catalytic performance of iron-modified zeolites, which have been documented widely in the literature [9,34,35], the introduction of Fe_2_O_3_ as the pillaring component seems to be an interesting idea to obtain an MCM-36-supported catalyst. What is more, so far, there are no reports in the literature on the introduction of iron oxide simultaneously with other pillars into the interlayer space of MCM-36.

Based on the studies on layered aluminosilicates, it can be assumed that potentially, the area of MCM-36 pillared with various metal oxides is not only limited to silica, alumina, titania, and alkaline metal oxides. Therefore, inspired by the possibility of the introduction of iron oxide pillars into the interlayer space of montmorillonite [32], we aimed to investigate whether Fe_2_O_3_ can act as a pillar of the layered structure of MCM-36. Additionally, our goal was to examine the difference between the physicochemical and catalytic properties of one-pot synthesized Fe-MCM-36 intercalated with SiO_2_, SiO_2_, and Al_2_O_3_. Last, but not least, the difference in the NH_3_-SCR catalytic performance of MCM-36 pillared with Fe_2_O_3_ and one-pot synthesized MCM-36 with Fe in the zeolitic framework was investigated and explained.

## 2. Results

### 2.1. Physicochemical Properties of the Materials

#### 2.1.1. Chemical Composition, Crystallinity, and Textural Characterization

The chemical composition of the pillared zeolites and their precursors was analyzed using ICP-OES. The obtained results, with regard to Si, Al, and Fe, as well as the real Si/Al molar ratios are presented in Table 1. It can be observed that the Si/Al molar ratio of the precursor was slightly lower than the intended one. Thus Al^3+^ cations were preferably built into the zeolitic framework of the material than Si^4+^ cations. After pillaring with SiO_2_, the content of silicon increased compared to the precursor, whereas the Si/Al molar ratio of the intercalated materials were determined by the type of pillars. One can notice that the value of this factor noticeably increased for M36-Si and FeM36-Si. In contrast, the simultaneous deposition of the SiO_2_–Al_2_O_3_ or SiO_2_–Al_2_O_3_-Fe_2_O_3_ pillars drastically decreased the Si/Al molar ratio due to the introduction of additional aluminum species from the intercalating solution. Importantly, the difference between the Al^3+^ content observed in MCM-22 (P) and FeM22 (P) suggested that some part of the aluminum cations was replaced in the zeolitic framework with Fe^3+^ cations. Interestingly, the content of iron in the material pillared with SiO_2_–Al_2_O_3_–Fe_2_O_3_ was found to be relatively close to that of the one-pot synthesized samples, which made the samples comparable with regard to their NH_3_-SCR catalytic activity.

**Figure 1:**
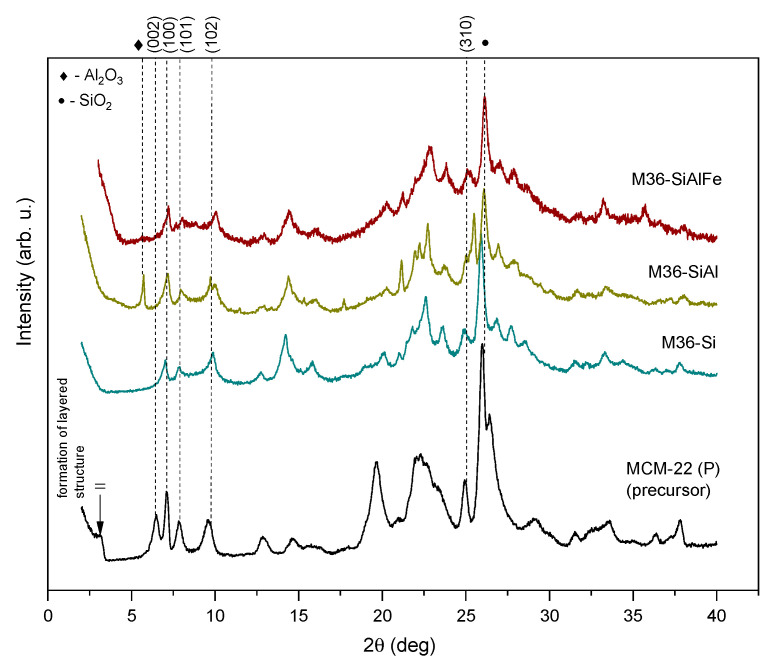
XRD diffractograms obtained from the MCM-22 (P) and M36 samples (black: MCM-22 (P); dark cyan: M36-Si; olive: M36-SiAl; wine: M36-SiAlFe).

The X-ray diffraction technique is a useful method which was utilized to investigate the characteristic layered and pillared structure, as well as the interlayer distance in the zeolites. The diffractograms of the precursor and MCM-36 intercalated with various pillars are presented in Figure 1. The reflections in the XRD pattern of MCM-22 (P), positioned at *2ϑ* ca. 6.4, 7.1, 7.9, 9.6, and 25° with the corresponding Miller indices of (002), (100), (101), (102), and (310), respectively, are typical for the zeolites of the MWW family [5]. Furthermore, the reflection present at *2ϑ* of about 3.1° was due to the formation of a layered structure of the precursor [36] and the (002) diffraction peak at *2ϑ* of ca. 6.5° reflected the regular separation between MWW layers, respectively [10]. Since the *d*-spacing of the reflection was 1.3 nm, the distance between two adjacent zeolitic layers ordered perpendicularly to the *c* axis was determined to approximately be 2.6 nm. Moreover, the well-defined doublet observed between 6.4 and 7.1° confirmed the formation of pure MCM-22 (P) instead of the 3D framework of MCM-49, which exhibited only one peak at 7.1°. The (100) and other diffraction maxima located at higher values of *2ϑ* confirmed the ordered arrangement of the zeolitic layers [13,37]. In the case of XRD patterns recorded for all of the pillared materials, the (002) reflections were absent, while the (100) peaks remained unchanged. Such results indicated that despite successful pillaring (proved by the disappearance of (002) reflection), the internal structure of the MWW layers determined by the (100) diffraction maximum was preserved. Another reason for the disappearance of the (002) reflection was delamination, and thus the loss of the perpendicular order of the layers with respect to the *c* axis, caused by the presence of the pillars [10]. Additionally, according to the study of Chmielarz et al. [38] on the alumina-intercalated vermiculites, the intense diffraction peak at *2ϑ* of ca. 5.8° in the XRD pattern of M36-SiAl appeared due to the successful pillaring with aluminum oxide. Furthermore, the lack of the additional reflection at around 6.7° confirmed that no alumina aggregates were deposited in the interlayer space of the material. Nonetheless, the diffraction maximum characteristic for Al_2_O_3_ pillars was absent in the case of M36-SiAlFe. This effect can be explained by the influence of Fe_2_O_3_ on the incorporation of alumina as either a pillar, or due to the exceptional dispersion of Al_2_O_3_ within the MWW layers. In addition, the XRD pattern of M36-SiAlFe was observed as the most distinct among all the pillared materials, and its shape further proved that it exhibited the highest dealumination degree.

**Figure 2:**
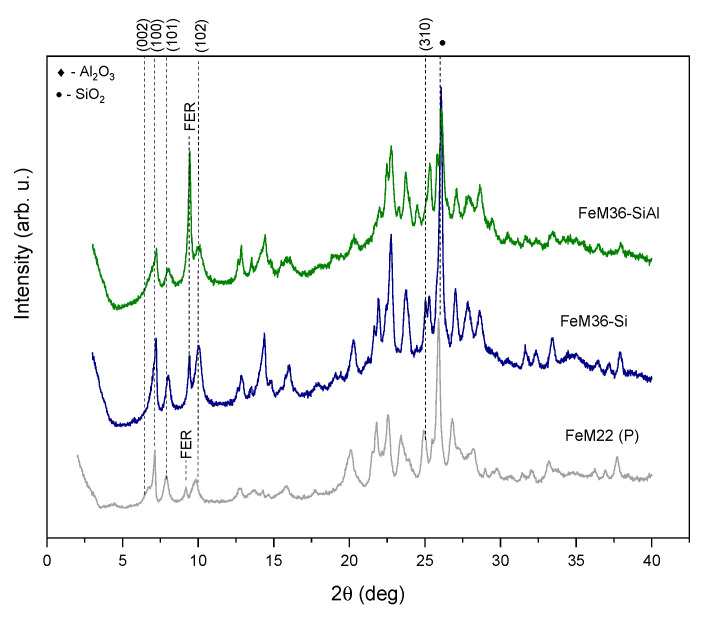
XRD diffractograms obtained from the FeMCM-22 (P) and FeM36 samples (gray: FeM22 (P); navy: FeM36-Si; olive: FeM36-SiAl).

The XRD patterns obtained for FeM36 samples, pillared with silica or silica and alumina and their precursor, are shown in Figure 2. It can be observed that FeM22 (P) exhibited diffraction peaks that are characteristic for MWW zeolites. However, the reflections observed were remarkably less intense compared to that of the iron-free precursor. The most significant difference was found in the case of the almost absent (002) diffraction maximum. Thereby the regular separation of the individual layers in the material was disturbed while Fe was introduced into the synthesis pot. Nevertheless, the appearance of the (100) and (101) reflections suggested that the presence of iron did not exhibit an influence on the internal structure of the MWW layers. The small diffraction peak observed at *2ϑ* of ca. 9.3° was attributed to the formation of the ferrierite (FER) phase in the zeolite [35,39]. On the other hand, both the low intensity of this maximum and other physicochemical features of the material further confirmed that the morphology and textural parameters are characteristic for the MWW zeolites. Additionally, the XRD patterns recorded for both FeM36 samples exhibited the typical (100) and (101) reflections, and due to partial delamination and rearrangement of the zeolitic layers, the (002) diffraction maximum disappeared following the pillaring process. However, in contrast to the precursor, the intensity of the diffraction maximum at 9.3° for the FeM36 samples was noticeably higher and increased with the increasing concentration of Al in the sample. Hence, the formation of FER was correlated with the aluminum content and can take place even after crystallization of the precursor.

**Figure 3:**
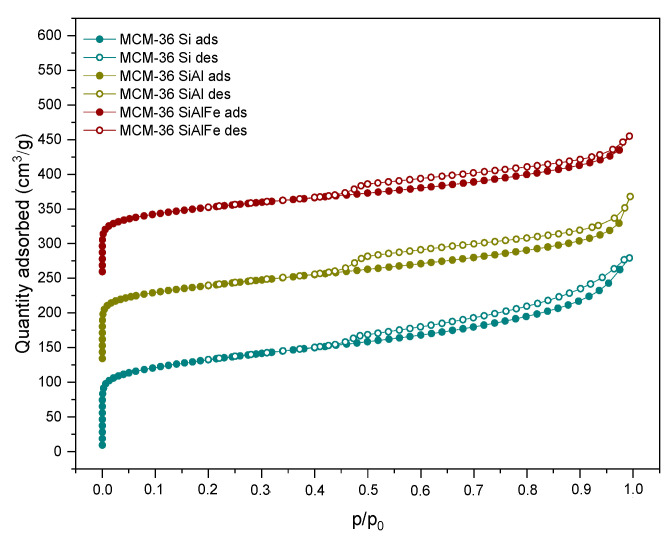
N_2_ adsorption–desorption isotherms of M36 samples.

Low-temperature N_2_ sorption experiments were performed to investigate the textural properties of the materials. The isotherms obtained for the M36 series of samples, presented in Figure 3, exhibited type II with the hysteresis loop beginning at *p*/*p_0_* = 0.45, and appearing due to the pillaring process. The hysteresis loop is of type H4, according to the IUPAC classification, which is characteristic for the aggregated crystals of zeolites and micro-mesoporous texture [40]. Regardless of the type of pillars, the isotherms observed were of a similar shape, clearly indicating the presence of the mesopores in the derivatives of MCM-36. Furthermore, the low, and gradually progressive increase in the N_2_ volume adsorbed in the micropores was caused by the pore-blocking pillars [5].

**Figure 4:**
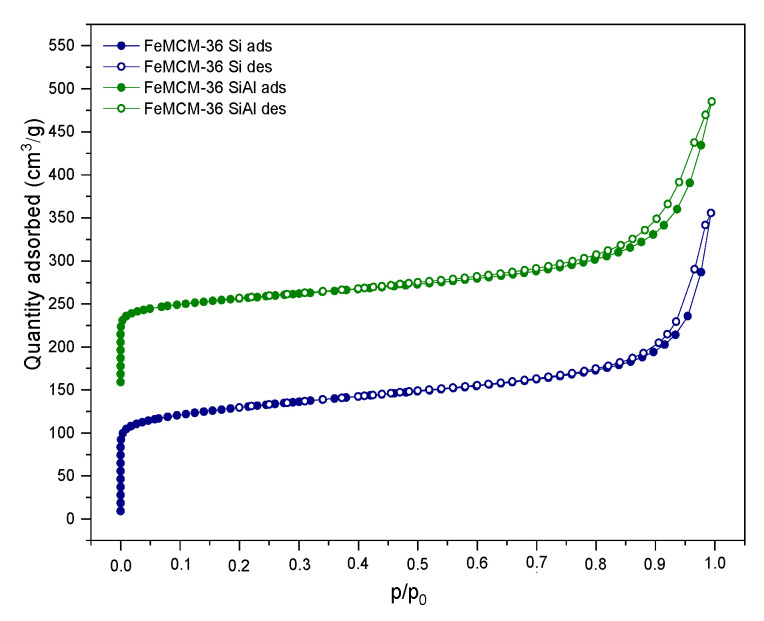
N_2_ adsorption–desorption isotherms of the FeM36 samples.

As presented in Figure 4, the introduction of iron into the synthesis pot remarkably changed the characterization of the N_2_ adsorption–desorption branches compared to the M36 samples (cf. Figure 3). The obtained isotherms resembled type II, with an H3 hysteresis loop, indicating a micro-mesoporous texture with narrow, slit-shaped pores and plate-like particles with voids present between the parallel layers. Additionally, the course of the hysteresis loop suggested that the zeolite consists of non-rigid aggregates rather than individual crystals [5,40]. Furthermore, regardless of the type of pillars, the isotherms of the FeM36 samples showed a very similar shape. However, a slightly lower condensation of nitrogen at a low *p*/*p_0_* detected for FeM36-SiAl can be attributed to the higher contribution of mesoporosity in the sample. In general, He et al. [41] distinguished two possible pathways of mesopore formation during the calcination of MCM-36. The first route assumes the formation of long, polymeric chains of silicon hydroxide in the interlayer space of the zeolite, followed by their expansion in two opposite directions. On the other hand, the organic molecules originating from the swelling solution are eliminated, leaving slots within the aluminosilicate framework. Therefore, the presence of SiO_2_ is generally regarded as the reason of the lower volume of nitrogen adsorbed at a low *p*/*p_0_*, compared to other layered zeolites [9]. Therefore, the simultaneous introduction of Al_2_O_3_ with SiO_2_ can be assumed as another factor limiting the access of nitrogen molecules to micropores and small mesopores.

The textural parameters of the analyzed materials were investigated based on the nitrogen sorption isotherms. The specific surface area (*S_BET_*) was determined with the BET method using adsorption data in the *p*/*p*_0_ range of 0.05–0.22, while the amount of N_2_ adsorbed at *p*/*p*_0_ = 0.98 corresponded to the total adsorption capacity. Hence, the total pore volume (*V_total_*) was calculated by converting the total amount of adsorbed nitrogen into its liquid volume, as recommended by Chlubná et al. [42]. The textural and structural parameters of the materials are shown in Table 2. The specific surface area of the M36 samples was between 363–475 m^2^·g^−1^, while for FeM36 it ranged between 392–476 m^2^·g^−1^, respectively. Furthermore, it can be observed that the total pore volume of the zeolites was dominated by the mesopores, as confirmed by the presence of the H3 and H4 hysteresis loops, which appeared in the isotherms. In the case of the M36 series, the *S_BET_* and total pore volume, as well as the area and volume of the micropores, all decreased, following the order of M36-SiAlFe < M36-SiAl < M36-SiAlFe, respectively. Such an effect was caused by the aggregation of pillars on the MWW layers, and consequently, pore blockage. The influence of the introduction of Fe into the synthesis pot was dependent on the type of pillars. In the case of FeM36-Si, the specific surface area and total pore volume increased significantly compared to the samples without iron. Therefore, iron was incorporated into the zeolitic structure, rather than being deposited in the form of bulky particles of Fe_2_O_3_ within the pore openings. Interestingly, for FeM36-SiAl the *S_BET_* and total pore volume were almost doubly lower than that of FeM36-Si. Such a result was deemed to be likely caused by the deposition of Al_2_O_3_ in all types of pores. Moreover, one can observe noticeable differences between the values of the specific surface area and pore volume of M36-SiAlFe and of the one-pot synthesized samples with Fe. The obtained results suggested that the incorporation of iron into the zeolitic framework was more beneficial for the development of porosity and for the specific surface area of the materials than the introduction of Fe_2_O_3_ in the form of catalytically active pillars.

#### 2.1.2. Morphology of the Materials

**Figure 5:**
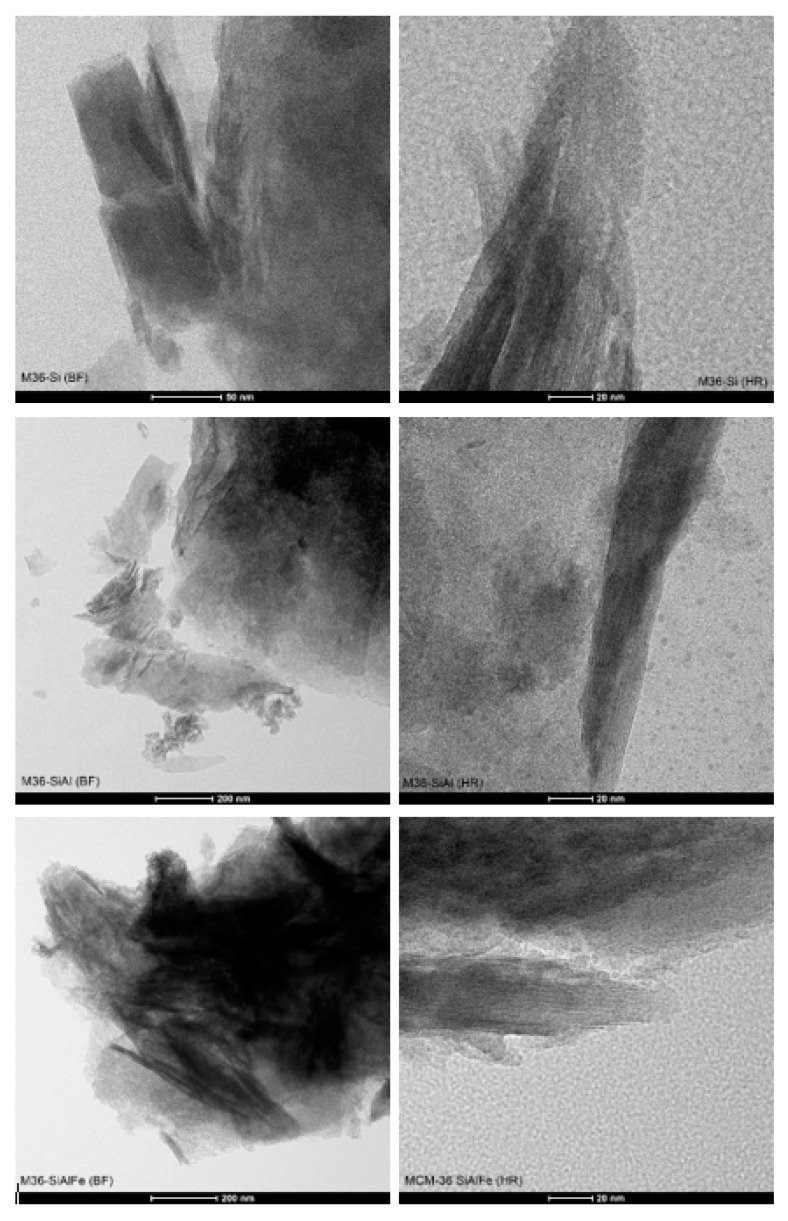

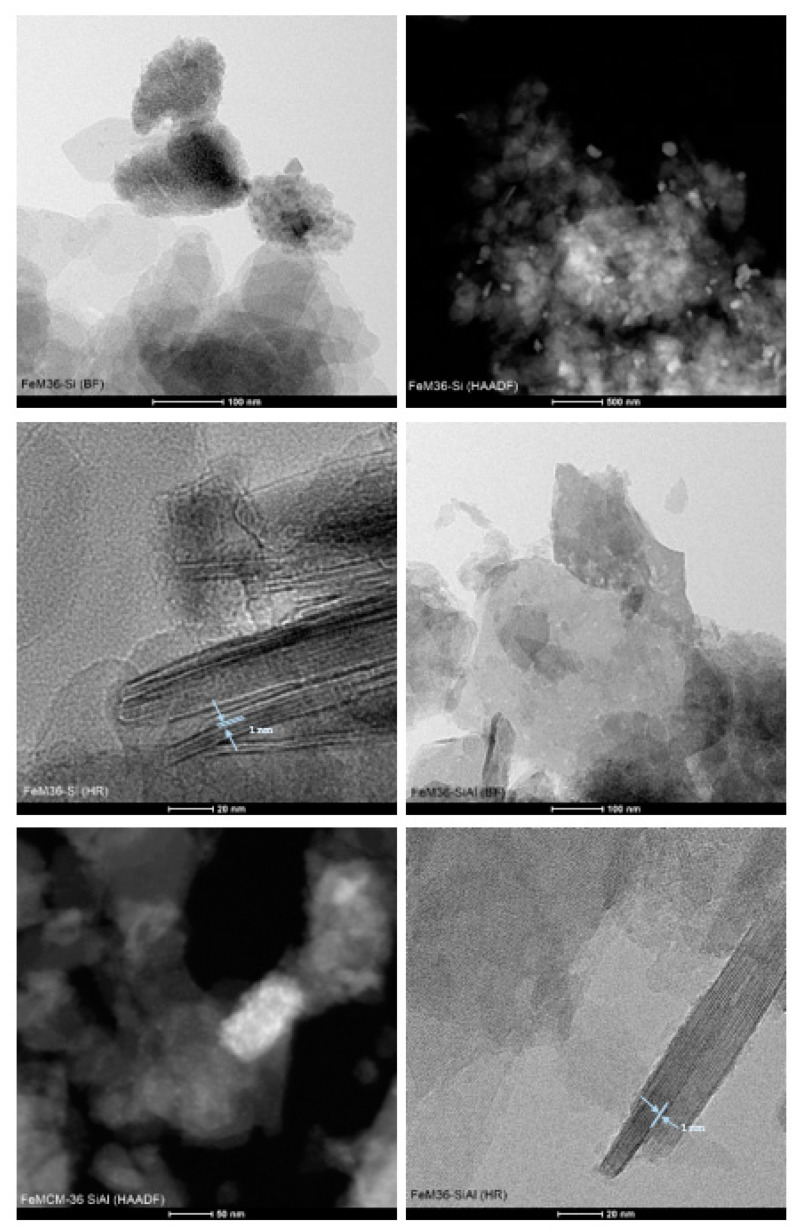
TEM images of the samples recorded in various modes.

The crystal morphology and layered structure of the samples on a nanoscopic scale was visualized using transmission electron microscopy. The micrographs presented in Figure 5 and Figure S1 were recorded in bright-field (BF), high-angle annular dark-field (HAADF), and high-resolution (HR) modes. According to Maheshwari et al. [43], the layered precursor, MCM-22 (P), is characterized by thin, circular disks, with the diameter between 500 and 1000 nm, and the thickness within 50–100 nm, respectively (images not presented). In general, all of the samples assessed exhibited a crystalline structure, which was retained after the swelling and pillaring processes. BF micrographs of the materials indicated that the crystals of MCM-36 have sharp facets and resemble a circular disk-like morphology. Furthermore, the dark bands separated by the white bands in the HRTEM images of the zeolites were assigned to the stacks of the long-range ordered MWW layers in the crystals. Therefore, regardless of the type of pillars or the presence of iron in the synthesis pot, all samples were successfully pillared. Nevertheless, it can observed that in the case of M36-SiAlFe, small domains of the stacked layers were surrounded by disordered amorphous domains. Therefore, the introduction of Fe_2_O_3_ most likely caused a partial destruction of the layered morphology of the zeolite. Interestingly, such an effect was not observed for M36-SiAl, suggesting that the influence of the intercalation procedure on the layered morphology was exclusively determined by the presence of iron oligocations in the pillaring solution. Moreover, one of the very important features determined by the means of TEM microscopy is the distribution of metallic species in the materials. As visualized in the BF and HAADF images, well-dispersed iron species were present in both FeM36-Si and FeM36-SiAl, which is consistent with the results of UV–Vis spectroscopy. Additionally, more bulky agglomerates of these iron species, with a size up to 100 nm, were also found in the materials. Nevertheless, as presented in the images, these metallic aggregates did not restrict the formation of the layered structure, nor in the successful introduction of the pillars.

#### 2.1.3. Characteristic Chemical Groups Present in the Materials

**Figure 6:**
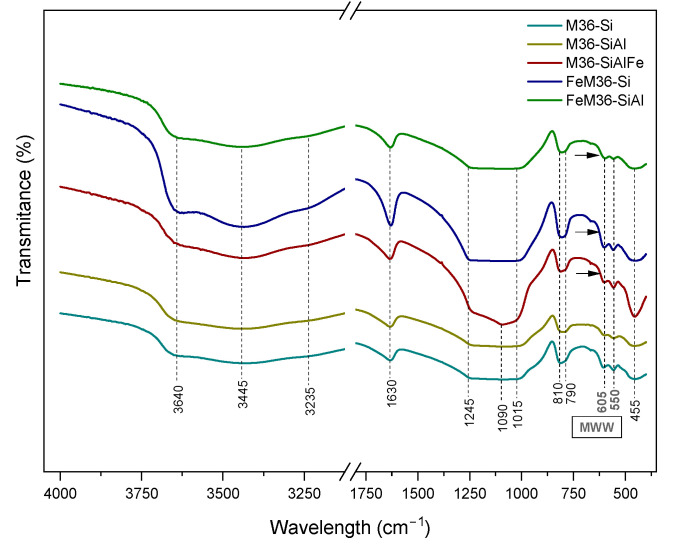
FT-IR spectra recorded for the M36 and FeM36 series of samples.

The characteristic chemical groups in the materials were analyzed using FT-IR spectroscopy and the obtained results are presented in Figure 6. In general, all of the spectra, regardless of the preparation procedure, exhibited the characteristic shape of the MWW zeolitic framework, and can be divided into two wavelength regions. The first region within 1300–400 cm^−1^ was determined to be related to the vibration modes of the aluminosilicate structure of the samples. The peaks appearing at 605 and 550 cm^−1^ corresponded to the double-six-ring (D6R) MWW topology [37,44], while the intense band at 455 cm^−1^ was related to the M-O bending vibrations (where M = Si, Al, or Fe) in the zeolites, respectively [13]. The latter peak was significantly more intense for M36-SiAlFe, which indicated the successful incorporation of Fe_2_O_3_ between the adjacent MWW layers [45]. Additionally, the broad band below 700 cm^−1^ (marked in the figure with arrows), which only appeared in the spectra of iron-modified samples, was deemed to be due to the stretching mode of Fe-O [45]. The peaks at 810 and 790 cm^−1^ can be assigned to the stretching vibrations of O-Si-O in SiO_4_^2−^ tetrahedra, respectively [46,47]. Furthermore, the band observed at 1090 cm^−1^ was due to the stretching modes of the internal M-O bonds in the MO_4_ tetrahedra [13]. The most distinct shape of this peak detected for M36-SiAlFe suggested that the introduction of three types of pillars could slightly change the internal interactions between the components of the framework. Additionally, the stretching bands of M-O-M were confirmed with the peak at 1245 cm^−1^ [47]. Last, but not least, the peak at 1630 cm^−1^ was determined to be related to water molecules physically bonded to the zeolitic framework [48]. The second characteristic region, with broad bands between 4000 and 3000 cm^−1^, respectively, indicated the presence of hydroxyl groups attached to the MWW framework [47]. Thus, the intense peak at 3640 cm^−1^ was attributed to Brönsted acid sites originating from Si(OH)Al in the supercages of the 10MR channels [49,50], while the one at 3445 cm^−1^ appeared due to the H-O-H stretching vibrations of the chemisorbed H_2_O molecules [51].

#### 2.1.4. Thermal Stability of the Materials

**Figure 7:**
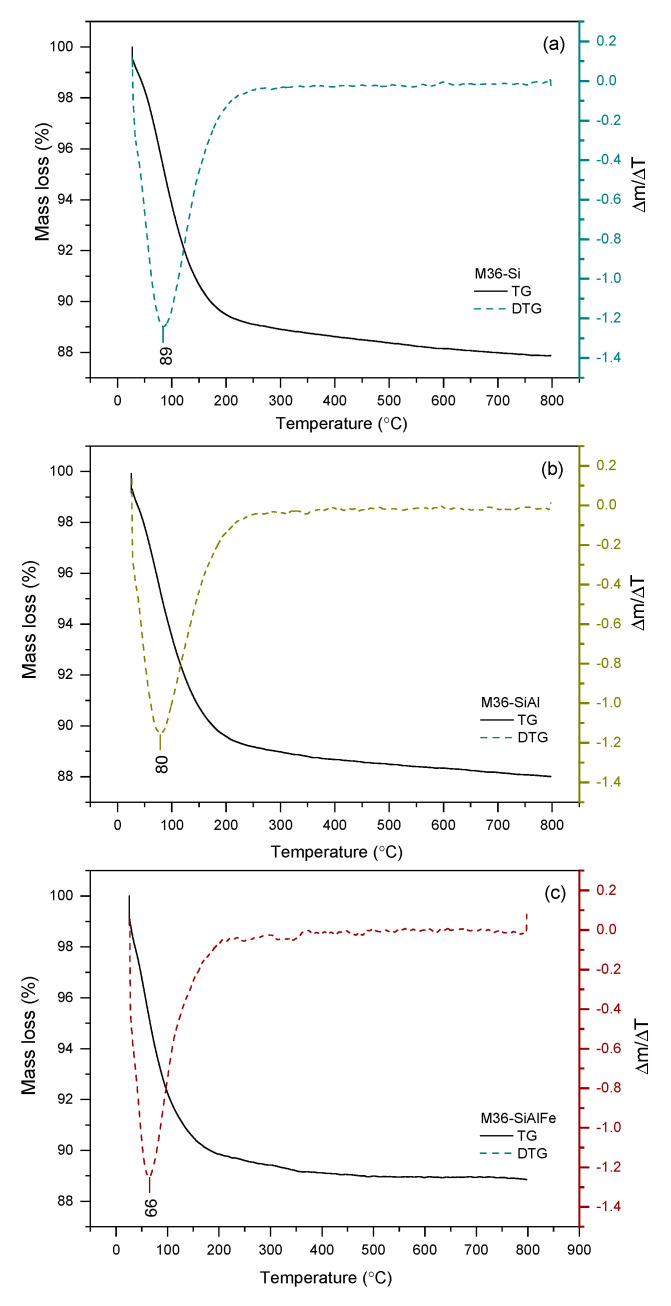
TGA and Δm/ΔT profiles of the M36 samples: (**a**) M36-Si; (**b**) M36-SiAl; (**c**) M36-SiAlFe.

**Figure 8:**
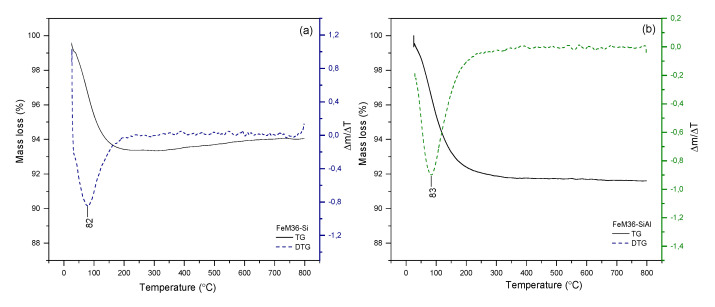
TGA and Δm/ΔT profiles of the FeM36 samples: (**a**) FeM36-Si; (**b**) FeM36-SiAl.

The thermal stability of the investigated materials was examined within 25–800 °C using thermogravimetric studies and performed in air at atmospheric pressure. The obtained thermograms are presented in Figure 7 and Figure 8 for the M36 and FeM36 samples, respectively. The percentage of total weight losses in the experimental temperature range is illustrated in Table 3. In general, all of the materials demonstrated a satisfactory thermal stability in the following order: M36-Si < M36-SiAl < M36-SiAlFe < FeM36-SiAl < FeM36-Si, and the weight loss at the analyzed temperature range did not exceed 12% for any of the samples. Additionally, the absence of a notable weight loss above 300 °C confirmed that the molecules of the structure-directing agents and surfactants used to prepare the materials were successfully removed during calcination. Interestingly, regardless of the type of material, only one peak, positioned below 100 °C, appeared in the Δ*m*/Δ*T* thermogram. The decrease in the weight exhibited in this temperature range was found to correspond to the removal of physically adsorbed moisture from the zeolitic structure [52]. Therefore, according to the data presented in Table 3, the introduction of additional metal oxides hindered the weight loss, as the pillars hampered the elimination of the water molecules from the inner structure of the zeolites. In the case of the M36 samples, there were also differences observed in the temperature of weight loss, which was in reverse to thermal stability: M36-SiAlFe < M36-SiAl < M36-Si. Additionally, for FeM36 samples, the decrease in the weight was remarkably lower compared to M36. Therefore, iron introduced directly into the zeolitic structure improved the thermal stability of the framework. Interestingly, the stabilization effect was stronger for the zeolites only intercalated with silica, which can be assigned to additional water molecules adsorbed on Al_2_O_3_ pillars. In contrast to M36 samples, the temperature of the weight loss for FeM36 was almost independent on the composition of intercalating particles.

#### 2.1.5. Acidic Properties of the Materials—NH_3_-TPD and Pyridine Adsorption Studies

**Figure 9:**
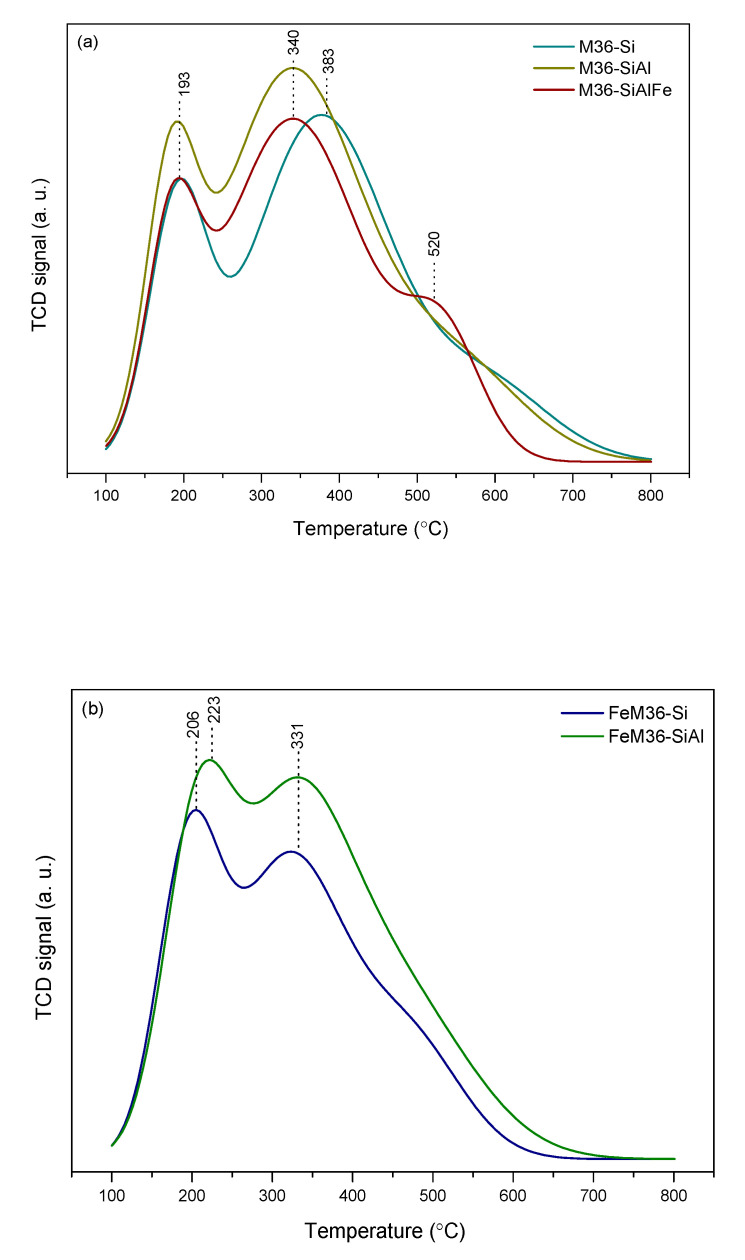
NH_3_-TPD profiles of the samples: M36 (**a**) and FeM36 (**b**).

The concentration of the acid sites in the materials was investigated using TPD with ammonia as a probe molecule. The shape of the desorption spectra provided important information on the strength and heterogeneity of the acid centers. The NH_3_-TPD profiles obtained for the M36 and FeM36 samples are presented in Figure 9, while the surface concentration and the ratio of Brönsted acid sites (BASs) to Lewis acid sites (LASs) (determined using Py-IR sorption studies) are shown in Table 4. In general, the NH_3_-TPD patterns of the samples consisted of two desorption maxima: low-temperature (LTM) and high-temperature (HTM), respectively, which corresponded to different types of sites, and were characterized by various acid strengths. The former peak positioned in the patterns within 147–250 °C was determined to be related to the desorption of NH_3_ weakly bonded to the acid centers. On the other hand, the latter, placed between 310–465 °C was deemed to be linked to the elimination of the strongly adsorbed ammonia moleculesIt can be observed from Figure 9a that for the M36 samples the temperature of NH_3_ desorption from the weak acid centers is independent of the type of the introduced pillars. However, intercalation of the material with Al_2_O_3_ or Al_2_O_3_-Fe_2_O_3_ shifted the HTM to a lower temperature. Nevertheless, despite the weaker strength of the strong sites, the introduction of alumina significantly elevated the total concentration of the acid centers in the zeolite (see Table 4). A similar effect was observed in terms of the influence of the Al_2_O_3_ pillars on the total concentration of the acid sites for the FeM36 samples. Additionally, the introduction of both silica and alumina into the interlayer space of the one-pot synthesized materials slightly shifted the LTM to a higher temperature range. Therefore, the presence of alumina not only increased the number of acid sites, but also intensified the strength of the bonding between NH_3_ and the surface of the zeolite. The impact of additional aluminum species in the samples on the acidic character of the materials is in line with that reported by Marosz et al. [10] for MCM-22, MCM-36, and ITQ-2 with various Si/Al molar ratios.

**Figure 10:**
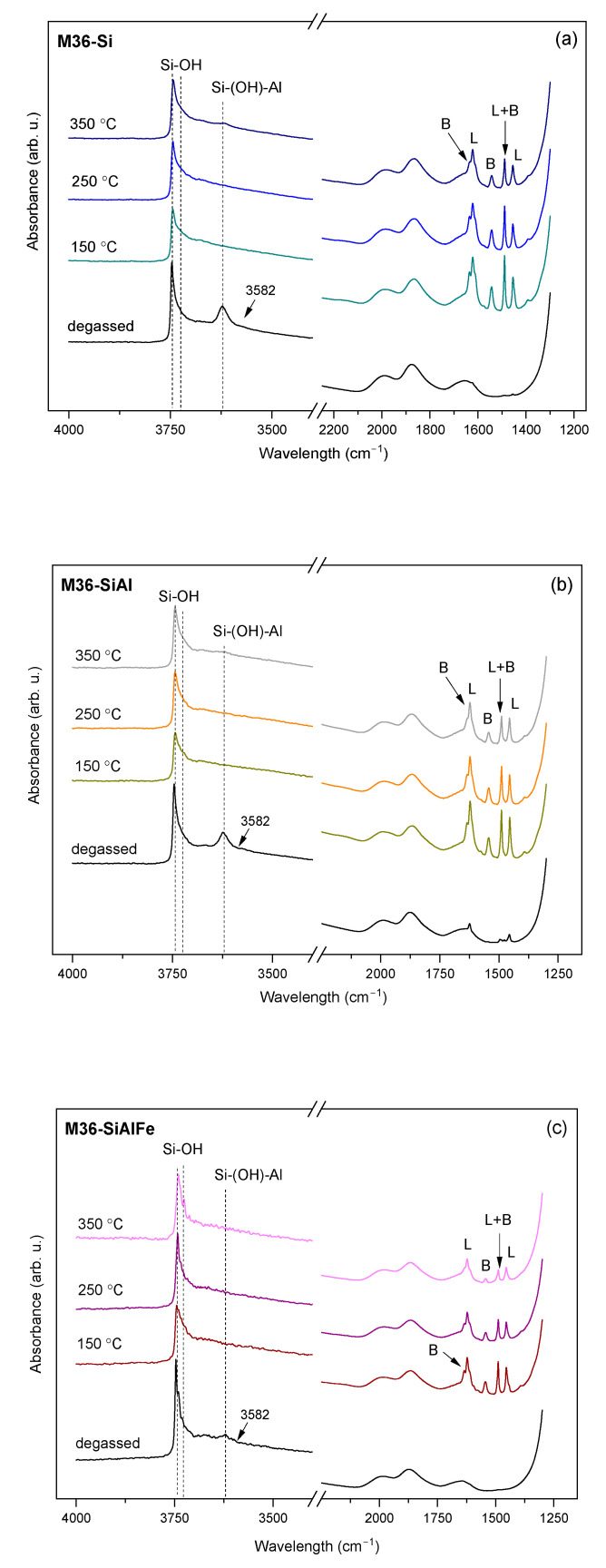
FT-IR spectra in the ν(OH) and Py ring vibrations regions of the M36 samples: (**a**) M36-Si; (**b**) M36-SiAl; (**c**) M36-SiAlFe (where B—Brönsted acid sites, L—Lewis acid sites).

**Figure 11:**
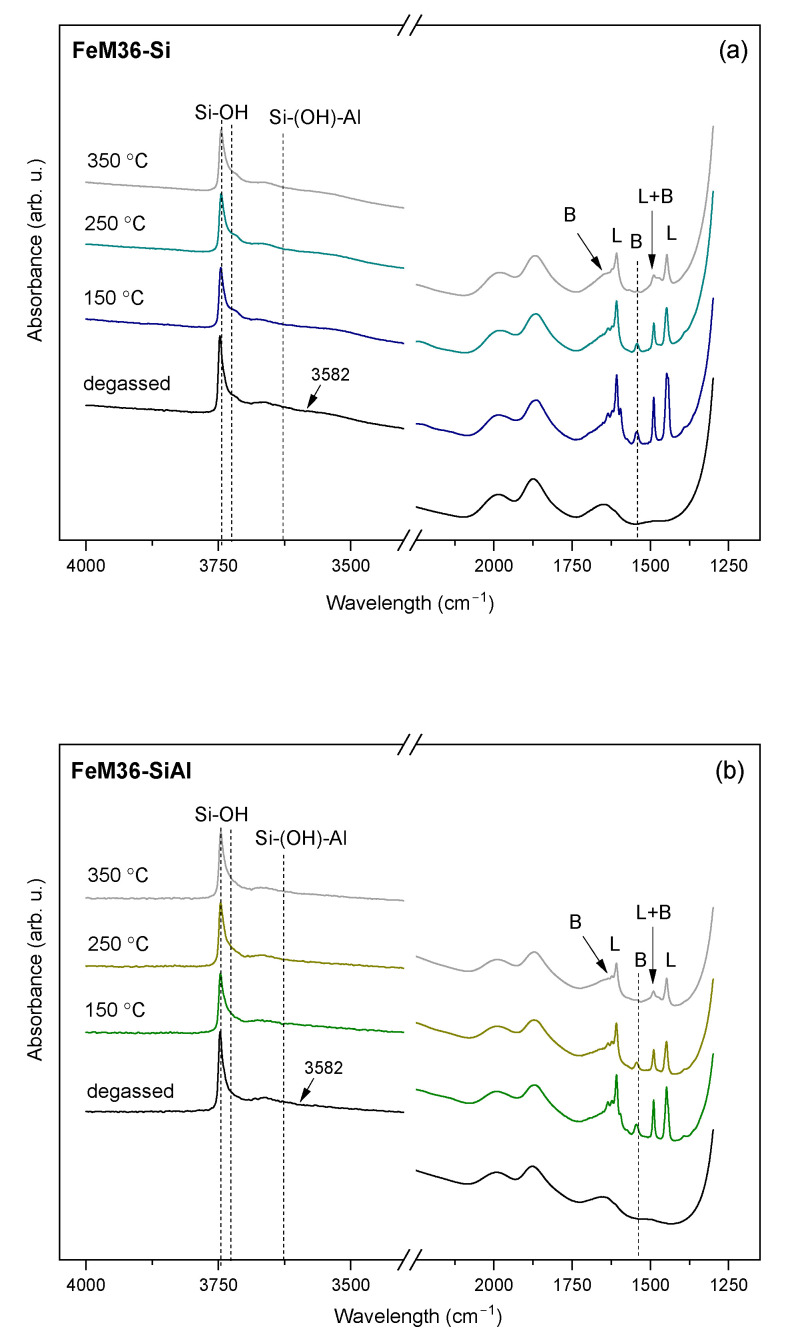
FT-IR spectra in the ν(OH) and Py ring vibrations regions of the FeM36 samples: (**a**) FeM36-Si; (**b**) FeM36-SiAl (where B—Brönsted acid sites, L—Lewis acid sites).

Despite the NH_3_-TPD experiment providing information on the total acidity of the materials, it cannot differentiate between the Brönsted and Lewis acid sites. Therefore, the contribution of the Brönsted (BASs) and Lewis (LASs) centers was investigated through the IR analysis of pyridine (Py) adsorption. The IR spectra in the *ν*(OH) and Py ring vibrations regions at 150, 250, and 350 °C are presented in Figure 10 and Figure 11 for the M36 and FeM36 samples, respectively. In general, hydroxyl groups in zeolites exhibit three characteristic bands in the *ν*(OH) region of the IR spectra: (1) terminal silanols at about 3750 cm^−1^, (2) H-bonded silanols at about 3743 cm^−1^, and (3) geminal silanols at about 3733 cm^−1^, respectively [53]. Thus, the strong band was placed at 3745 cm^−1^, and the shoulder at ca. 3730 cm^−1^ can be assigned to the isolated external and geminal internal Si-OH groups, respectively [54]. A slightly reduced intensity of the band at 3745 cm^−1^ was detected for all of the samples after the adsorption of Py. Therefore, external Si-OH groups participated in the generation of PyH^+^ ions (BAS) [5]. Furthermore, based on the silanol frequencies of zeolite Y and ZSM-5, Corma et al. [55] assigned the broad band in the region of 3740–3500 cm^−1^ to hydrogen-bonded silanol groups, which are associated with the local framework vacancies. Additionally, according to Gil et al. [53], the intense band present at about 3620 cm^−1^ corresponds to the presence of acidic bridging Si-(OH)-Al in 12-MR and 10-MR channels, while the very broad peak observed at a of low intensity at 3582 cm^−1^ is related to the OH groups located in hexagonal prisms in supercages. It can be observed that these bands appeared only in the case of the materials without iron. Therefore, the formation of the hydroxyl nests characteristic for the MWW framework could be interrupted after the introduction of Fe into the zeolitic structure. Moreover, after the chemisorption of pyridine, the band at 3582 cm^−1^ disappeared, which was deemed to most likely be due to the interaction of OH groups in the hexagonal prisms with Py molecules, which subsequently withdrew protons from their original position [56]. Additionally, the weak band, placed within 3670–3660 cm^−1^, was more pronounced for the samples with iron, and is characteristic for OH groups located on the extraframework Al sites [42].

During the adsorption of Py, the band at ca. 3620 cm^−1^, which was assigned to the OH groups bridged between the framework of the Si and Al atoms [57] completely vanished in favor of the appearance of the new bands in the range of 1700–1400 cm^−1^. Typically, Py adsorbed on LASs shows absorption bands within 1633–1600 cm^−1^, about 1580 cm^−1^, 1503–1488 cm^−1^, and 1460–1447 cm^−1^, respectively; on the contrary, BASs can be identified in the Py-IR spectra by the peaks located at about 1640 cm^−1^, 1540 cm^−1^, and between 1500 and 1485 cm^−1^, respectively. Thereby, the bands at 1640 cm^−1^ and 1545 cm^−1^ appeared in all of the spectra due to the formation of PyH^+^ species (BAS), while those at 1616 cm^−1^ and 1450 cm^−1^ corresponded to coordinatively-bonded pyridine (LAS), respectively. Furthermore, the band placed at about 1483 cm^−1^ was characteristic for both the Brönsted and Lewis acid centers [5,58]. The intensity of the specific peaks was dependent on the presence of iron, since Fe^3+^ introduced into the structure modified the acidic nature of the samples by the replacement of Brönsted with Lewis acid centers. For example, among the M36 samples, the peak at 1640 cm^−1^ related to BASs exhibited the lowest intensity in the case of M36-SiAlFe, as a result of the contribution of Fe^3+^ to Lewis-type acidity. What is more, for FeM36 materials the band was almost absent, which indicated a successful incorporation of the iron cations into the MWW structure. Only small changes in the intensity of the peaks related to Lewis acidity during the evacuation of pyridine within 150–350 °C suggested the very high strength of the centers originating from aluminum and iron in the zeolitic framework.

The concentrations of Brönsted and Lewis acid sites calculated for the investigated materials are shown in Table 4. The total densities of the centers evaluated by Py-IR were remarkably lower comparing to the values obtained from NH_3_-TPD. Chen et al. [35] explained this effect with the different accessibilities of the ammonia and pyridine molecules to the inner porous structure of the MWW zeolites. Since the kinetic diameter of Py (0.5 nm [53]) is greater than that of NH_3_ (0.26 nm [59]), 10-MR pore openings of the MWW framework (0.41 × 0.51 nm or 0.4 × 0.55 nm, respectively [60]) are barely accessible for pyridine molecules. In contrast to pyridine, ammonia used as a probe molecule in the TPD measurement did not encounter any diffusion limitations. However, the trend observed in the total densities of the acid centers in the samples determined by Py-IR was determined to be in agreement with that of NH_3_-TPD.

What is important, as detailed in Table 4, is that the accessibility of the pyridine molecules oscillated around 49–65%, and strongly depended on the chemical composition of the zeolites. Considering the FeM36 samples, apart from the framework aluminum cations, iron species also contributed to the total acidity of the materials. However, despite Lewis acid centers having being delivered by Fe^3+^, pore-blocking polynuclear Fe_x_O_y_, possibly generated during formation of the zeolitic framework, limited the access of Py to the acidic adsorption centers. A similar effect related to the presence of transition metal in MCM-36 was observed by Jankowska et al. [13]. In contrast, in the case of the M36 samples, the accessibility followed the order of M36-SiAl < M36-Si < M36-SiAlFe, which clearly indicates that despite lower values of textural parameters (see Table 2), iron oxide pillars were beneficial for basic molecules to approach the acid centers.

In general, the results presented in Table 4 revealed a strong relationship between the chemical composition of the materials and the density of the specific acid centers. In the case of M36-Si, the number of BASs was slightly higher comparing to the LASs. However, a significant increase in Lewis-type acidity was observed after the introduction of Al or Al and Fe species. Such an effect was related to the acidic nature of the isolated Fe^3+^ cations and Al^3+^ in Al_2_O_3_ [61,62]. On the other hand, the concentration of BASs remarkably decreased, which was due to the fact that one LAS related to Fe^3+^ was generated in favor of three BASs [5]. Additionally, Lewis acid sites could be formed by iron-containing oligomers, which is especially possible for M36-SiAlFe and FeM36-SiAl, since these samples were the most abundant in this kind of species, as confirmed by the UV–Vis results. Additionally, among the zeolites with Fe, the concentration of BASs was the highest for M36-SiAlFe. Such a result can be explained by the study of Hu et al. [63], who reported that Brönsted acid sites are located on the surface of FeO_x_ aggregates in Fe-modified ZSM-5. Hence, the significantly higher density of BASs in M36-SiAlFe compared to FeM36 samples most likely resulted most from the speciation of iron in the material.

The changes in the total concentration of Py chemisorbed on the Brönsted and Lewis acid sites caused by the temperature treatment of the pre-adsorbed materials are presented in Figure S2. Typically, the number of pyridine molecules attached to the acid centers gradually reduced with the increasing temperature. Additionally, the experiment revealed a significant difference between the strength of the acid sites in the materials, since both the number and the strength of the Brönsted acid sites in FeM36 was noticeably lower than that in the M36 group. Such a result can be assigned to a lower electronegativity of Fe(OH)Si (present in the one-pot synthesized samples) compared to Al(OH)Si, and thus, the weaker acidity of the materials [64]. What is more, as already mentioned, is that not only the strength, but also the concentration of the acid sites in FeM36 was lower than in M36. Additionally, for all of the samples except for M36-Si, the strength and concentration of the LASs were higher than those of BASs. Moreover, Lewis acid sites were remarkably stronger while aluminum or iron were incorporated in the form of pillars. In general, the acidic character of the zeolites is related to the position of aluminum in the zeolitic structure. According to Palčić and Valtchev [65], Lewis acid sites developed on the surface or as an extraframework species exhibit a stronger strength compared to the framework moieties. Therefore, the energy of the bonding between the probe molecule and the acid sites was higher in the case of the Fe_2_O_3_ and/or Al_2_O_3_-pillared samples.

Last, but not least, the densities of the Brönsted and Lewis acid sites in the samples in relation to their strength are depicted in Figure S3. In the case of the M36 series of samples, the proportion of acid sites of specific type was similar for M36-Si and M36-SiAl. However, the introduction of Fe_2_O_3_ as a pillar led to the replacement of the strong Brönsted sites with the centers of medium strength. Additionally, the concentration of strong and weak Lewis sites in M36-SiAlFe was similar to that of M36-SiAl. Hence, the high density of LASs in both samples was determined to be due to the presence of extraframework aluminum, which originated from the Al_2_O_3_ pillars. In contrast to the Fe_2_O_3_-pillared sample, modification of the zeolite framework with Fe^3+^ species resulted in the complete removal of the strong Brönsted acid sites along with the considerable reduction in the number of medium-strength sites. Moreover, one can observe the opposite proportion of weak and strong Lewis sites in the M36 and FeM36 series of samples. This result can be related to the different positions of the aluminum species in the zeolitic framework, or to the various speciation of iron in the iron oxide-pillared and one-pot synthesized samples.

#### 2.1.6. Distribution of Iron in the Materials

**Figure 12:**
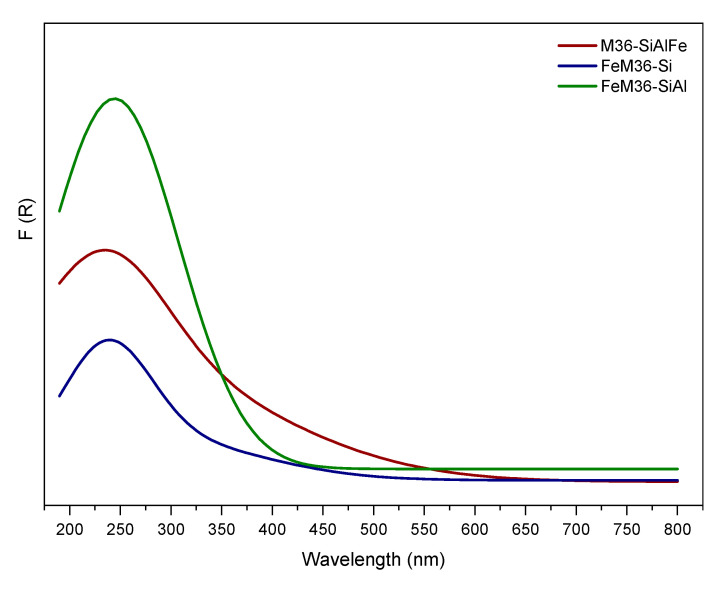
UV–Vis DR spectra of MCM-36 with iron introduced in the form of pillars or with a one-pot synthesis.

UV–Vis DR experiments were performed over the samples modified with iron to determine the form and aggregation of the metal species present in the zeolites. The obtained results, presented in Figure 12, indicated that all of the materials exhibited strong absorption bands below 400 nm. The region between 220 and 230 nm corresponded to the charge-transfer transition involving monomeric Fe^3+^ in a tetrahedral coordination (FeO_4_), while the band within 260–270 nm was ascribed to the presence of Fe^3+^ in an octahedral coordination, respectively [66]. Furthermore, the increased intensity in the wavelength region of 350–400 nm, detected for M36-SiAlFe and FeM36-SiAl, corresponded to the octahedral Fe^3+^ cations in small oligomeric clusters of Fe_x_O_y_ [67]. Additionally, the peak observed between 400 and 550 nm appearing in the spectrum of M36-SiAlFe proved the presence of small iron oxide crystallites [5], which is in line with the results presented in the TEM micrographs. Importantly, these species were absent in the spectra obtained for FeM36 samples. Hence, these results confirmed that the introduction of iron cations into the synthesis pot favored the deposition of monomeric Fe^3+^ and partially prevented the formation of bulky iron oxide species. Moreover, the spectra of FeM36 samples clearly showed that simultaneous pillaring with SiO_2_ and Al_2_O_3_ promoted the deposition of the active phase in the form of isolated cations, which is in full agreement with the form of iron determined from the TEM micrographs.

### 2.2. NH_3_-SCR Catalytic Performance of the Materials

**Figure 13:**
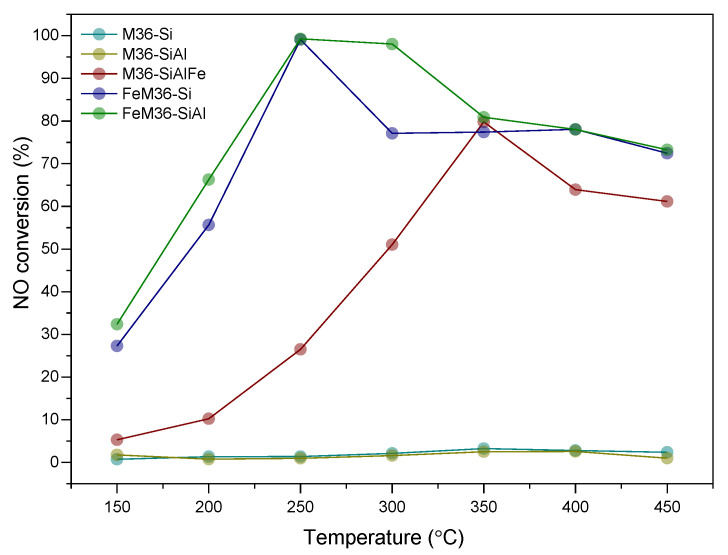
NO conversion obtained for the samples.

All of the analyzed materials were assessed as catalysts for the selective catalytic reduction with ammonia, which aimed to transform NO to the mixture of N_2_ and H_2_O, while N_2_O is a by-product of the process. Figure 13 shows a strong correlation between the presence of iron and the NO conversion obtained for the materials. One can note that M36-Si and M36-SiAl were completely inactive in the catalytic reaction. In contrast, the introduction of Fe_2_O_3_ pillars enhanced the activity of M36-SiAlFe in the entire investigated temperature range. Furthermore, it can be observed that NO reduction increased for the sample linearly with temperature, which is characteristic for Fe-modified zeolites [68,69,70]. The temperature of 50% conversion (*t*_50_) for M36-SiAlFe was 295 °C, while the maximum NO reduction of 78% was obtained at 350 °C, respectively. The decreased activity of the material above 350 °C indicates the occurrence of a few side reactions, such as the oxidation of ammonia, which has been previously reported in the literature for the pillared clays modified with iron [38]. The non-desired reactions involved in the catalytic process have generally been attributed to the presence of more aggregated iron species, which generally promote the oxidation of ammonia [71]. One of the possible pathways of NH_3_ oxidation involves the generation of N_2_O, described by reactions (1)–(3):

4NO + 4NH_3_ + 3O_2_ → 4N_2_O + 6H_2_O (1)

2NH_3_ + 2O_2_ → N_2_O + 3H_2_O (2)

2NH_3_ + 4NO → 2N_2_ + N_2_O + 3H_2_O (3)

**Figure 14:**
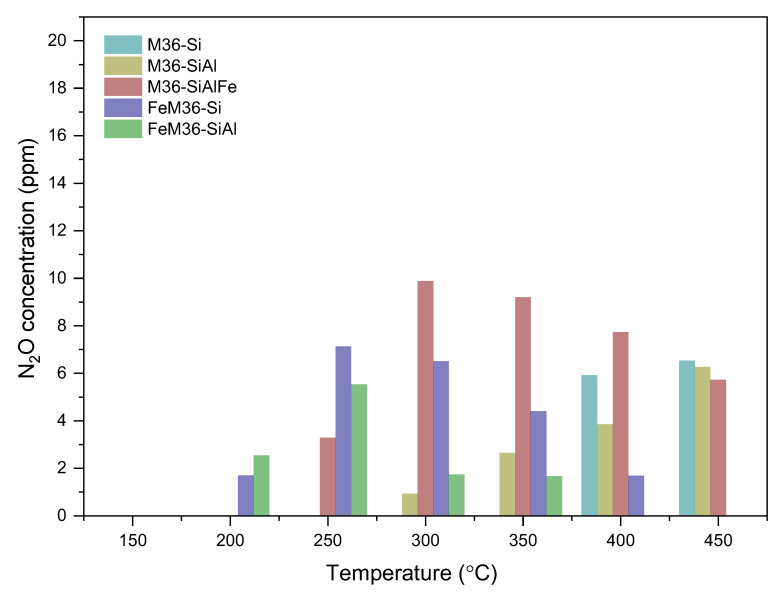
Concentration of N_2_O emitted during the catalytic reaction over the samples.

The occurrence of the processes (1)–(3) was confirmed with the drastically increased concentration of nitrous oxide in the post-reaction gas mixture above 300 °C, as illustrated in Figure 14. Notably, reactions (1) and (3) involve NO, and thereby, its concentration in the exhaust gas should be theoretically lower. In fact, within 300–350 °C, the catalytic activity of the sample exhibited an increasing trend. However, between 350 and 450 °C, NO reduction was gradually lowered to be subsequently maintained at a constant level within 400–450 °C. Therefore, the contribution of reactions (1)–(3) to N_2_O generation above 300 °C can be assumed to be temperature-dependent.

As presented in Figure 13, the introduction of iron through the one-pot synthesis method dramatically improved the low-temperature activity of the zeolite. In the case of FeM36-Si, 50% of the NO conversion was reached at 193 °C, which was slightly higher than that of FeM36-SiAl, for which *t_50_* was 174 °C, respectively. Furthermore, both the one-pot synthesized samples exhibited a 100% of NO reduction at 250 °C. The complete conversion of nitrogen oxide was maintained up to 300 °C for FeM36-SiAl. In contrast, for FeM36-Si the activity gradually dropped to 77% above 250 °C, and was sustained till 450 °C. The outstanding low-temperature activity of the materials can be assigned to the presence of isolated, monomeric Fe^3+^ sites, whose abundance was evidenced by the UV–Vis experiments. According to Brandenberger et al. [72], these sites are characterized with a lower activation energy compared to polynuclear species, and thus contribute to NO conversion below 300 °C. What is more, the remarkably higher activity of FeM36-SiAl, which was most pronounced between 250–350 °C, was determined to be related to the presence of Al_2_O_3_ pillars, which exhibited a high capacity to chemisorb ammonia molecules, especially at high temperatures [73]. Thus, there was a significant activation effect related to the simultaneous introduction of SiO_2_ and Al_2_O_3_ pillars into one-pot synthesized Fe-MCM-36. On the one hand, some parts of NH_3_ could interact with NO according to the Eley–Rideal reaction mechanism, a characteristic for high-temperature region, to yield N_2_ and H_2_O. On the other side, Chmielarz et al. [71] suggested that the acid sites delivered by alumina activate NH_3_ for its direct oxidation, rather than for the DeNO_x_ process. This postulate was supported by the activity drop detected for FeM36-SiAl above 300 °C. Alternatively, the decreased conversion of NO in the high-temperature region could have resulted from the low concentration of polynuclear iron species, which showed a higher activation energy and contribute to catalytic activity above 250 °C.

The generation of nitrous oxide at the reactor outlet is one of the crucial factors monitored during the NH_3_-SCR reaction. The negligible concentration of the emitted N_2_O obtained for the FeM36 samples (below 12 ppm), combined with the decreased catalytic activity above 300 °C suggested that the most probable product of NH_3_ oxidation is NO. One can observe that the formation of N_2_O for the one-pot synthesized samples reached its maximum at 250 °C, and progressively decreased above this temperature. Additionally, it was remarkably higher for FeM36-Si. According to Koebel et al. [74], the production of nitrous oxide during NH_3_-SCR is related to the formation of ammonium nitrate (NH_4_NO_3_) on the catalyst surface and its further decomposition to N_2_O and H_2_O below 260 °C, which can be described by the reaction (4):

NH_4_NO_3_ → N_2_O + 2H_2_O (4)

What is interesting, is that regardless of the production of nitrous oxide through reaction (4), the concentration of the by-product in the exhaust gas decreased for both samples to finally reach the level below the detection limit at 450 °C. Based on the study of Delahay et al. [75], N_2_O formed during the reaction could be reduced by ammonia to form N_2_ and H_2_O, as according to reaction (5):

2NH_3_ + 3N_2_O → 4N_2_ + 3H_2_O (5)

The occurrence of reaction (5) was also confirmed by the reduced activity of the samples, which could have resulted from the consumption of the reducing agent as a consequence of its non-selective reaction with N_2_O.

## 3. Discussion

Overall, the results of our study revealed that MCM-36 belonging to the MWW zeolite family can be prepared using various pillars, including the mixtures of SiO_2_, Al_2_O_3_, and Fe_2_O_3_, respectively. Moreover, some of the atoms incorporated into the aluminosilicate framework can be successfully substituted with Fe^3+^, which considerably activates the material in the NH_3_-SCR reaction. Importantly, regardless of the introduction procedure, all of the samples were characterized with a similar content of iron, as confirmed with the ICP-OES studies (cf. Table 1). It was found from XRD studies that MCM-36 can be intercalated with various pillars, and that no secondary phases were formed in the case of M36 series. However, the arrangement of the MWW layers was dependent on the type of pillars, since both XRD and TEM studies indicated that the simultaneous introduction of silica, alumina, and iron oxide resulted in the highest delamination degree. On the other hand, it was observed that incorporation of iron into the synthesis pot not only resulted in the generation of small and well-dispersed aggregated clusters (cf. Figure 5), but also resulted in the formation of the secondary FER phase. Ben Younes et al. [76] investigated the effects of the structure of Fe-zeolites on their catalytic performance in NH_3_-SCR. According to the authors, in the case of FER, the small aperture of channels resulted in the limited access of the reacting molecules to the inner structure of the material, and thus, led to a very poor activity in NO reduction. Therefore, a positive contribution of the FER phase on the high catalytic activity of FeM36 samples can be excluded. Additionally, TG studies indicated that the incorporation of iron into the framework stabilized the zeolitic structure, which was more pronounced for FeM36-Si. Such an effect was correlated with the hampered diffusion of H_2_O molecules from the pores, caused by the presence of SiO_2_ pillars. Furthermore, low-temperature N_2_ sorption studies indicated the dominance of mesopores in the materials. In contrast, the formation of microporosity in the samples was considerably limited by the type of pillars incorporated in the material. Both the volume and the area of micropores decreased (following the order of M36-SiAlFe < M36-SiAl <M36 Si), due to the fact that the pores below 2 nm were able to be completely clogged by the additional Al_2_O_3_ and Al_2_O_3_-Fe_2_O_3_ pillars. Additionally, relatively similar textural characterizations of M36-Si and FeM36-Si confirmed that the incorporation of iron into the framework generated mainly isolated framework Fe^3+^ cations, which is consistent with the results of UV–Vis spectroscopy. Furthermore, considering the porosity and structural parameters, one-pot synthesis was remarkably more beneficial compared to the introduction of Fe_2_O_3_ pillars. Additionally, the dispersion of iron in the FeM36 samples was strongly correlated with the type of interlayer species, since the presence of SiO_2_–Al_2_O_3_ resulted in the formation of individual clusters which were visible in the TEM micrographs.

What particularly stands out from our studies is that despite the diffusion limits, the introduced pillars created several new adsorption centers for the molecules reacting during the NH_3_-SCR process. The investigation of the acidic character of the materials indicated that regardless of the preparation procedure, Al_2_O_3_ pillars significantly increased the number of acid sites. However, as confirmed by the NH_3_-TPD studies, in contrast to the FeM36 samples, the simultaneous introduction of silica and alumina weakened the strength of the acid centers of the M36 materials. What was also noticeable, was that the density of acid sites was not a key factor in the good catalytic performance of the zeolites, since M36-Si and M36-SiAl (most abundant in acidic centers) were completely inactive in NH_3_-SCR. Thus, the relatively satisfactory NO conversion obtained for M36-SiAlFe can be exclusively ascribed to the presence of iron oxide pillars, while the acid sites originating from Al_2_O_3,_ and the aluminosilicate framework only acted as a reservoir of ammonia molecules. The experimental studies on the mechanism of NH_3_-SCR indicated that the primary step of the process is the adsorption and activation of NH_3_ on the catalyst surface [77,78,79], and according to Wang et al. [80], Lewis acid sites can adsorb ammonia more actively than the Brönsted centers. Therefore, the activation of NH_3_ and the catalytic activity of M36-SiAlFe was related to the newly generated adsorption sites, originating from Fe^3+^ delivered by the Fe_2_O_3_ pillars. Additionally, since all the M36 samples exhibited a relatively similar density of the Lewis acid centers, the considerably increased conversion of NO resulted from the outstanding redox features of the iron species. In fact, Long and Yang [81] postulated that ammonia adsorbed on the Brönsted sites in a form of NH_4_^+^ cations is more stable at high temperatures compared to NH_3_ coordinated on the Lewis centers. However, according to Schwidder et al. [82], Brönsted acidity is dispensable to obtain a satisfactory conversion of NO over Fe-exchanged zeolites within 250–600 °C. Amores et al. [83] completely excluded the involvement of NH_4_^+^ ions in the standard NH_3_-SCR, while Brandenberger et al. [84] assumed that Brönsted acid sites are not required for the adsorption and activation of ammonia. On the contrary, Liu et al. [85] found no evidence for the superiority of Brönsted or Lewis acid sites in the activation of the ammonia and NH_3_-SCR performance. Nevertheless, the authors postulated that all of the acid centers in the support act as a reservoir for the reducing agent, which is then transported to the Fe species for the reaction with NO. Therefore, not the acidic, but redox character of Fe^3+^ present in the Fe_2_O_3_ pillars of M36-SiAlFe contribute to the superior catalytic activity of the material [86]. Additionally, the poor NO adsorption capacity of Fe^3+^ combined with the temperature window of 300–450 °C suggested that the reaction followed the Eley–Rideal mechanism, which is characteristic for the high-temperature region of NH_3_-SCR [80,87]. Our findings are in line with the studies performed by Apostolescu and co-workers [88] on the NH_3_-SCR catalytic potential of Fe_2_O_3_-based materials. According to the authors, iron sites participate in the dissociative adsorption of ammonia, which results in the formation of reactive amide surface species along with the partial reduction of iron. The amide moieties then react with gas-phase NO and form NO and H_2_O, while the iron center is re-oxidized by O_2_ to complete the redox cycle. Apart from the moderately good high-temperature performance of M36-SiAlFe, one can observe that the emission of N_2_O for the material was the highest among all of the samples. Such a result was related to the side reaction of NH_3_ oxidation, which is typically involved in the Eley–Rideal mechanism [89]. Additionally, the generation of nitrous oxide for the sample can be a consequence of the diffusion limits set by the pore-locking pillars, which led to non-desired interactions between the reacting molecules.

In contrast to M36-SiAlFe, the group of Fe-modified one-pot synthesized samples exhibited very high NO reduction rates below 300 °C. The observed increase was deemed to be certainly due to the modified redox and acidic nature of the materials caused by the deposition of various iron species into the zeolitic framework. In our previous study [9], we confirmed that the outstanding low-temperature activity of silica-intercalated MCM-36 with iron incorporated directly into the zeolitic framework was attributed to the presence of the isolated Fe^3+^ species. However, the studies reported here indicated that the temperature window of the material can be expanded by the incorporation of Al_2_O_3_ simultaneously with the silica pillars. Taking into consideration the decreased value of the specific surface area and the textural parameters of FeM36-SiAl, the superior catalytic performance of the material compared to FeM36-Si was undoubtedly related to the co-existence of SiO_2_ and Al_2_O_3_ between the adjacent MWW layers. One of the possible reasons of the promoting effect of alumina on the expansion of the temperature window is the presence of the coordinatively unsaturated Al^3+^ ions. These sites generated additional Lewis acid sites, which was confirmed by the Py-IR studies. Another possible explanation of the higher NO conversion obtained for FeM36-SiAl is related to the type of iron sites present in the material. As determined from the UV–Vis spectra, the sample pillared with Al_2_O_3_ was not only abundant in the monomeric Fe^3+^, active in the low-temperature region, but also contained a considerable amount of polynuclear moieties, which typically contribute to NO conversion within 300–400 °C [72]. Moreover, the TEM micrographs recorded for the samples a revealed better dispersion of the metallic species in FeM36-SiAl. Therefore, it is possible that alumina could prevent the migration of Fe^3+^ from the framework positions, which resulted in the formation of catalytically inactive, bulky aggregates. On the other hand, the study studies of Chmielarz et al. [38] proved that alumina pillars introduced into the interlayer space of aluminosilicates significantly contributed to the oxidation of ammonia to nitrogen and water vapor. Thus, despite the positive influence on the temperature window, the promotion of the non-selective reactions of NH_3_-SCR by the Al_2_O_3_ pillars cannot be completely excluded.

One can observe that the amount of N_2_O produced within 250–350 °C was noticeably higher for FeM36-Si compared to FeM36-SiAl. Such a result can be linked with the fact that FeM36-SiAl contained a remarkably higher number of monomeric sites (see UV–Vis studies). Since it was confirmed that these species are inactive for ammonia oxidation of up to 500 °C [72], the difference between the emission of N_2_O noticed for FeM36-Si and FeM36-SiAl was deemed to be related to the speciation of iron in the samples.

Based on the results of the catalytic studies, it can be concluded that the higher conversion of NO obtained for one-pot synthesized materials compared to Fe_2_O_3_-pillared sample resulted from the compilation of the specific features of the zeolites. In fact, the NH_3_-TPD studies revealed that both types of materials were characterized with a similar density of the acid centers, and an approximate number of the Lewis acid sites. Nevertheless, the number of Brönsted acid sites was found to be considerably lower in the case of the FeM36 samples, which thereby confirmed the theory that BASs are not essential for NH_3_-SCR. Additionally, since the one-pot synthesized materials exhibited a lower concentration of strong Lewis sites, it can be assumed that not the strength, but the ratio of the BAS/LAS was the crucial acidity-related factor determining the catalytic performance of the materials. Furthermore, the structural and textural characterizations suggested that the introduction of the active phase in the form of pillars limited the development of microporosity due to pore clogging, caused by the deposition of bulky oxide clusters. In contrast, the direct incorporation of iron into the zeolitic framework not only eliminated the problem of aggregation, but also placed the metal species into their catalytically active monomeric form.

## 4. Materials and Methods

### 4.1. Preparation of the Materils

#### 4.1.1. Synthesis of the Precursor MCM-22 (P)

The precursor of the MCM-36 zeolite, with the intended Si/Al molar ratio of 25, was obtained according to the procedure reported by Corma et al. [90]. The molar composition of the synthesis gel was SiO_2_: 0.02 Al_2_O_3_: 0.5 HMI: 0.09 NaOH: 45 H_2_O, and the mixture was prepared using sodium hydroxide (NaOH, Scharlab, Barcelona, Spain), sodium meta-aluminate (NaAlO_2_, 56% Al_2_O_3_, 37% Na_2_O, Carlo Erba Reagents, Milan, Italy), silica (Aerosil 200, Evonik Rohm GmbH, Essen, Germany), hexamethyleneimine (HMI, 98 wt.%, Scharlab, Barcelona, Spain), and deionized water. The typical preparation procedure involved dissolving 0.375 g of NaOH and 0.375 g of NaAlO_3_ in 81.71 g of deionized water and stirring the mixture for 15 min at room temperature. After that time, 6 g of silica was slowly added to the solution and was maintained under stirring. Afterwards, 4.96 g of HMI was introduced into the solution dropwise, and the gel was mixed for 2 h. The resulting slurry was subsequently crystallized in a stainless-steel autoclave lined with polytetrafluoroethylene (PTFE), under rotation (60 rpm) at 150 °C for 7 days. The resulting solid was filtered, washed several times to neutral pH, and dried overnight at 100 °C in air. The obtained precursor was labeled as **MCM-22 (P).**

#### 4.1.2. Swelling of MCM-22 (P)

The swelling procedure, aiming to expand the interlayer space of MCM-22 (P), was performed using a mixture of cetyltrimethylammonium bromide (CTMA^+^Br^-^) and tetrapropylammonium bromide (TPA^+^Br^−^). The bromide ions were partially exchanged (ca. 70% and ca. 30% for CTMA and TPA, respectively) for hydroxide anions, using a hydroxide form of Amberlite IRN78 (strong anion exchange resin). Firstly, 5 g of MCM-22 (P) was added into the mixture of 100 g CTMA^+^Br^−^/OH^−^, 30 g of TPA^+^Br^-^/OH^-^, and 30 g of deionized water. The slurry was then stirred at 80 °C for 16 h under reflux. Secondly, it was filtered, washed with a small amount of water, and dried overnight at 60 °C. The obtained solid product was used to synthesize MCM-36 intercalated with SiO_2_, SiO_2_–Al_2_O_3_, and SiO_2_–Al_2_O_3_–Fe_2_O_3_ pillars.

#### 4.1.3. Intercalation with Various Pillars

In order to prepare MCM-36 pillared with SiO_2_, the swollen MCM-22 was mixed with tetraethyl orthosilicate (TEOS, 98%, Aldrich, Spain) as the precursor of silica. The weight ratio of the solid to the pillaring agent was 1:5, and its suspension was stirred at 90 °C for 25 h in N_2_ atmosphere. Afterwards, the mixture was filtered, and the pillared solid was washed with ethanol. Subsequently, the material was dried overnight at 60 °C in air, and then calcined at 540 °C in N_2_ atmosphere for 1 h and in air for 16 h, respectively. The prepared sample was labeled as **M36-Si.**

MCM-36 pillared with SiO_2_ and Al_2_O_3_ was prepared using the pillaring solution obtained according to the reproduced methodology proposed by Barth et al. [20]. Typically, 250 cm^3^ of 0.2 M NaOH solution was added dropwise to 250 cm^3^ of Al(NO_3_)_3_ solution (0.1 M, Scharlab, Barcelona, Spain) under stirring. The solution was agitated at 70 °C for 4 h and subsequently aged at room temperature for 72 h. The introduction of silica pillars into the interlayer space of MCM-22 (P) was performed analogously to **M36-Si**, while intercalation with Al_2_O_3_ was carried out by the hydrolysis of the SiO_2_-pillared sample as suspension in the water (weight ratio 1:10) with the pillaring solution of alumina pillars at 40 °C for 6 h. The volume of the pillaring solution was adjusted to introduce 24 mmol of Al^3+^/1 g of the sample pillared with silica. The desired pH of the pillaring process (~8.0) was controlled using NaOH. The resulting material was then filtered, dried at 60 °C in air, and calcined at 540 °C in N_2_ atmosphere for 1 h and in air for 16 h, respectively. The zeolite intercalated with silica and alumina was labeled as **M36-SiAl.**

MCM-36 pillared with SiO_2_, Al_2_O_3_, and Fe_2_O_3_ was synthesized using the pillaring solution that was prepared according to the reproduced method reported by Muñoz et al. [91]. Firstly, 500 cm^3^ of the solution of Fe^3+^ and Al^3+^ ions (derived from iron and aluminum nitrate, respectively) was prepared in such a way that Fe^3+^/(Fe^3+^ + Al^3+^) = 0.2, while the total concentration of trivalent cations (Me^3+^) was 0.4 M, respectively. Secondly, the sufficient quantity of 0.4 M NaOH solution was slowly added to the continuously stirred mixture to obtain an OH^−^/Me^3+^ molar ratio of 2.0. The resulting liquid, clear product was aged at room temperature for 72 h. The pillaring procedure resembled that of SiO_2_–Al_2_O_3_. However, the volume of the intercalating mixture was adjusted to introduce 20 mmol of Me^3+^/1 g of the sample pillared with silica. The resulting material was then filtered, dried at 60 °C in air, and calcined at 540 °C in N_2_ atmosphere for 1 h and in air for 16 h, respectively. The zeolite intercalated with silica, alumina, and iron oxide was labeled as **M36-SiAlFe.**

The series of MCM-36 intercalated with various pillars was collectively labeled as **M36**.

#### 4.1.4. One-Pot Synthesis of Fe-MCM-36 Intercalated with Silica or Silica-Alumina Pillars

The one-pot synthesis of the layered precursor with Fe^3+^ introduced directly into the zeolitic framework was conducted based on the reproduced methodology suggested by Chen et al. [35]. The material with the molar composition of SiO_2_: 0.017 Al_2_O_3_: 0.05 Fe(NO_3_)_3_: 0.5 HMI: 0.4 NaOH: 45 H_2_O (Si/Al molar ratio of ca. 28, Si/Fe molar ratio of ca. 20) was prepared as follows: 1.10 g of NaOH (Scharlab, Barcelona, Spain), 0.31 g of NaAlO_2_ (NaAlO_2_, 56% Al_2_O_3_, 37% Na_2_O, Carlo Erba Reagents, Milan, Italy), and 1.64 g of Fe(NO_3_)_3_·9 H_2_O (Sigma Aldrich, Merck, KGaA, Darmstadt, Germany), respectively were dissolved in 57 g of deionized water and stirred at room temperature for 30 min. Subsequently, 11.85 g of silica sol (40.5 wt.%, Sigma Aldrich, Merck, KGaA, Darmstadt, Germany) was added dropwise to the mixture. Afterwards, 3.97 g of HMI (98 wt.%, Scharlab, Barcelona, Spain) was slowly introduced into the solution, which was then homogenized under stirring for the next 2 h. Finally, the prepared gel was crystallized in a Teflon-lined stainless-steel autoclave rotated at 150 °C for 7 days. After that time, the solid product was filtered, washed several times with distilled water to a neutral pH, and dried overnight at 100 °C. The synthesized solid product was subsequently labeled as **FeM22 (P).** Prior pillaring with silica or silica-alumina oxides, FeM22 (P) was swollen according to an identical procedure to that of the iron-free samples. Similarly, the synthesis of one-pot synthesized MCM-36 with Fe^3+^ in the structure, intercalated with silica or silica and alumina was conducted analogously to the iron-free MCM-36. The obtained materials were labeled as **FeM36-Si** and **FeM36-SiAl** for the silica- and silica-alumina-intercalated samples, respectively.

The series of one-pot synthesized Fe-MCM-36 intercalated with various pillars was collectively labeled as **FeM36**.

### 4.2. Physicochemical Characterization of the Materials

The content of Si, Al, and Fe in the samples was determined using inductively coupled plasma optical mass spectroscopy (ICP-OES, QTEGRA). Crystalline structure and phase purity were analyzed using X-ray powder diffraction (XRD). The XRD patterns were collected using a Panalytical Empyrean diffractometer with a Cu Kα radiation source (λ = 1.54184 Å) at a tube current of 40 mA and a voltage of 40 kV in the *2θ* scanning range of 2–40°, respectively. XRD data were complied using X’pert HighScore software (with the database). Low-temperature N_2_ sorption measurements were performed using the Micromeritics 3Flex Surface Characterization gas adsorption analyzer. Prior to each experiment, the samples were degassed under vacuum at 90 °C for 1 h and then at 350 °C for 5 h. The specific surface area (*S_BET_*) of the materials was calculated according to the BET (Brunauer–Emmet–Teller) equation from the adsorption branch, as recommended by Rouquerol [92]. The external surface area, along with the volume and surface area of the micropores were calculated using the Harkins and Jura model (*t*-plot analysis). The mesopore volume was determined using the BJH model from the adsorption branch of the nitrogen isotherm. Transmission electron microscopy (TEM) was performed using a Tecnai TF20 X-TWIN (FEG) microscope (Thermo Fisher Scientific) equipped with an EDS detector (EDAX), working at an accelerating voltage of 200 kV. Samples for TEM observations were prepared by the drop-casting of powder/isopropyl alcohol suspensions on carbon-coated copper TEM grids. Thermogravimetric and differential thermal analyses (TGA-DTA) were conducted using Mettler Toledo TGA/SDTA 851E equipment. The experimental procedure consisted of progressive increases in the temperature to quantify the weight losses, Typically, the investigated sample was heated to 800 °C with a temperature ramp of 10 °C·min^−1^ and using an air stream of 20 cm^3^·min^−1^, respectively. The concentration and strength of the acid sites was determined by the temperature-programmed desorption of NH_3_ (NH_3_-TPD) using Autochem II (Micrometrics) apparatus. The experiments were performed in the temperature range of 100–800 °C in a fixed bed continuous flow microreactor. Prior to the measurement, each sample was pre-treated in a stream of Ar at 100 °C for 1 h. Afterwards, the materials were equilibrated at 100 °C in a stream of He and were saturated for 30 min in a flow of 1 vol.% of NH_3_ in He. Subsequently, the samples were heated up to 800 °C with a temperature ramp of 10 °C·min^−1^ in an Ar stream. The desorbed amount of ammonia was analyzed by the means of a thermal conductivity detector (TCD) and coupled GC-MS mass spectrometer (OmniStar, Bazers Instruments). The NH_3_ uptake was calculated from the amount of desorbed gas volumes from the area under the NH_3_-TPD curve. The setup was calibrated prior to analysis with the known amounts of ammonia to determine the precise area of a single pulse registered by the TCD detector. Infrared spectroscopic monitoring of pyridine sorption on the samples’ surfaces enabled one to distinguish between the Brönsted acid sites (BAS) and Lewis acid sites (LAS). To evaluate the contribution of BASs and LASs, self-supported wafers (*φ* = 10 mg·cm^−2^) of calcined samples, previously activated at 400 °C and 10^−2^ Pa overnight in a Pyrex vacuum cell, were contacted with 6.5·10^2^ Pa of pyridine vapor at room temperature. Afterwards, the probe molecule was desorbed in vacuum at increasing temperatures (150, 250, and 350 °C, respectively). The spectra were recorded at room temperature and were scaled according to the sample weight (10 mg). The concentrations of BASs and LASs were determined according to the methodology reported previously by Emeis [93]. The total number of BASs and LASs in the samples was calculated from Py-IR spectra recorded at 150 °C using the following absorption coefficients: *ε* (BAS) = 1.67 cm·µmol^−1^, *ε* (LAS) = 2.22 cm·µmol^−1^, and the intensities of the corresponding Py absorption maxima (1545 cm^−1^ for BASs and 1445 cm^−1^ for LASs, respectively). The quantities of BASs and LASs were estimated using Equations (6) and (7), respectively:

(6) $$\mathrm{BAS}=\varepsilon {\left(\mathrm{BAS}\right)}^{-1}\;\times \;\mathrm{IA}\;(B)\;\times \;\frac{\pi R^{2}}{W}$$

(7) $$\mathrm{LAS}=\varepsilon {\left(\mathrm{LAS}\right)}^{-1}\;\times \;\mathrm{IA}\;(L)\;\times \;\frac{\pi R^{2}}{W}$$

where *C* is the concentration of BAS or LAS (µmol·g^−1^), *ε* is the integrated molar extinction coefficient (cm·µmol^−1^), IA is the integrated absorbance (cm^−1^), *R* is the radius of the wafer used for the measurement (cm), and *W* (g) is the weight of the sample. The strength of the BASs and the LASs were defined as follows: the strong sites were calculated from Py-IR spectra recorded at 350 °C, the medium sites were determined from the difference of Py-IR spectra recorded at 350 and 250 °C, and the weak sites were estimated from the difference of Py-IR spectra recorded at 250 and 150 °C, respectively. The characteristic chemical groups present in the zeolitic frameworks of were assessed using FT-IR spectroscopy. The spectra were collected using the Perkin Elmer Frontier spectrometer in the wavelength region of 4000–400 cm^−1^ with a resolution of 4 cm^−1^. The coordination and aggregation of iron species introduced into the zeolites were analyzed by the means of ultraviolet diffuse reflectance spectroscopy (UV–Vis DR). These experiments were conducted using a Cary 5 spectrophotometer equipped with a diffuse reflectance accessory. The spectra were recorded in the wavelength range of 200–800 nm, with a resolution of 2 nm.

### 4.3. Catalytic Tests

NH_3_-SCR catalytic tests were conducted over the samples under atmospheric pressure in a fixed-bed flow microreactor with a quartz tube. Typically, 200 mg of the material was outgassed at 400 °C for 30 min in a flow of nitrogen and cooled to 100 °C. The model gas mixture consisted of 800 ppm of NO, 800 ppm of NH_3_, 3.5 vol.% of O_2_, and He as an inert, respectively and the total gas flow was 100 cm^3^·min^−1^, which led to a GHSV of 30,000 cm^3^·h^−1^·g^−1^. The experiments were performed in the temperature range of 150–400 °C with 50 °C as a step. The concentrations of residual NO and N_2_O (the side-product of the reaction) in the outlet gas were continuously monitored with a FT-IR detector (ABB 2000, AO series). The results of the catalytic tests were calculated based on the specific algorithm and were collected in a form of .xls file. Such a manner of measurement did not provide FT-IR spectra of the post-reaction gas mixture; however, it enabled the relatively fast calculation of the experimental results. The catalytic activity, expressed as the conversion of NO, was calculated using Equation (8):

(8) $$NO_{conv}=\frac{\left(C_{NO_{in}\;}-C_{NO_{out}}\right)}{C_{NO_{in}\;}}\times 100\%$$

where $NO_{conv}$—NO conversion, $C_{NO_{in}—}$inlet concentration of NO, and $C_{NO_{out}—}$outlet concentration of NO in the gas mixture, respectively.

## 5. Conclusions

The results of our study proved that MCM-36 can be successfully prepared using not only silica, but also with its mixture with Al_2_O_3_ or Al_2_O_3_–Fe_2_O_3_. Additionally, the latter material exhibited a relatively satisfactory performance as the NH_3_-SCR catalyst, due to the introduction of iron and the modification of the acidic nature of the zeolite. Furthermore, it was found that Al_2_O_3_ and Fe_2_O_3_ acted synergistically in the catalytic reaction, since both Al^3+^ and Fe^3+^ delivered active centers for the reaction to proceed. However, the introduction of iron in the form of metal oxide pillars did not provide enough isolated framework species, which contributed to a low-temperature activity in NH_3_-SCR. The one-pot synthesized material intercalated with SiO_2_–Al_2_O_3_ was the most active sample with a 100% of NO conversion having being maintained within 250–300 °C. The superior performance of the catalyst over the SiO_2_-pillared one was ascribed to a higher acidity; however, it cannot be excluded that alumina promoted the distribution of iron in the zeolitic framework and prevented the migration of the monomeric framework Fe^3+^ to the extraframework positions. The gradually decreasing activity of the Fe-modified samples in the high-temperature range was attributed to the side reaction of ammonia oxidation. Nonetheless, the negligible concentration of N_2_O registered during this process indicated that NH_3_ was most likely transformed into molecular nitrogen.

In summary, these conducted studies demonstrate that iron can be introduced into the MWW structure using diversified methods, which can further determine the speciation of the metallic phase, the acidic character, and the catalytic performance in NH_3_-SCR. The presented research significantly contributes to the area of novel modifications in the MWW zeolites and opens a new path in the facile synthesis of novel NH_3_-SCR catalysts.

## Figures and Tables

**Table 1:** Chemical composition of the materials determined using ICP-OES.

Sample Code	Si (wt%)	Al (wt%)	Fe (wt%)	Si/Al
MCM-22 (P)	33.23	1.41	0	23.0
M36-Si	39.47	2.08	0	29.0
M36-SiAl	36.58	4.74	0	7.5
M36-SiAlFe	34.01	6.09	5.21	5.4
FeM22 (P)	32.72	1.91	4.96	21.0
FeM36-Si	34.02	1.16	5.02	28.0
FeM36-SiAl	37.59	3.54	4.84	10.0

**Table 2:** Textural properties of the materials determined by low-temperature N_2_ sorption.

Sample Code	S_BET_ *^a^* (m^2^·g^−1^)	External Surface Area *^b^* (m^2^·g^−1^)	Micropore Area *^b^* (m^2^·g^−1^)	Total Pore Volume *^c^* (m^3^·g^−1^)	Micropore Volume *^b^* (m^3^·g^−1^)	Meso + Macropore Volume *^d^* (m^3^·g^−1^)
MCM-22 (P)	569	141	434	0.480	0.172	0.308
M36-Si	410	213	197	0.315	0.084	0.231
M36-SiAl	363	203	160	0.285	0.069	0.216
M36-SiAlFe	716	507	209	0.740	0.162	0.578
FeM22 (P)	392	147	245	0.438	0.100	0.338
FeM36-Si	569	141	434	0.480	0.172	0.308
FeM36-SiAl	410	213	197	0.315	0.084	0.231

*^a^* Specific surface area determined using the BET method. *^b^* External surface area, micropore area, and micropore volume analyzed by *t*-plot. *^c^* Total pore volume at *p*/*p*_0_ = 0.98. *^d^* V_micro+meso_ = V_total_ − V_micro_.

**Table 3:** Total weight losses recorded for the samples during TG analysis.

Sample Code	Weight Loss (%)
M36-Si	11.57
M36-SiAl	11.17
M36-SiAlFe	10.03
FeM36-Si	5.66
FeM36-SiAl	7.91

**Table 4:** Density of acid sites in the materials determined using NH_3_-TPD and IR studies after chemisorption of Py.

Sample Code	Acid Site Density from NH_3_-TPD (µmol·g^−1^) *^a^*	Acid Site Density from Py-IR (µmol·g^−1^)				Accessibility *^c^* (%)
		Total *^b^*	BASs *^b^*	LASs *^b^*	%BAS/%LAS	
M36-Si	1511	912	485	426	53/47	60
M36-SiAl	1609	878	386	492	44/56	55
M36-SiAlFe	1225	794	338	456	43/57	65
FeM36-Si	1253	570	112	458	20/80	45
FeM36-SiAl	1326	651	164	488	25/75	49

*^a^* Total density of the acid sites determined by NH_3_-TPD experiments. *^b^* Total density of Brönsted (BASs) and Lewis (LASs) acid sites calculated from the Py-IR spectra recorded at 150 °C. *^c^* The accessibility of Py to acid sites was defined as the ratio of the total acid sites calculated from Py-IR to that determined from NH_3_-TPD.

## Data Availability

Data are contained within the article.

## Supplementary Materials

The following supporting information can be downloaded at: https://www.mdpi.com/article/10.3390/molecules28134960/s1, Figure S1: TEM images of the samples recorded in various modes.; Figure S2: Changes in the concentration of pyridine adsorbed on the acid centers of the materials at 150, 250, and 350 °C.; Figure S3: Densities of Brönsted (BASs) and Lewis (LASs) acid sites in relation to their strength: strong, medium, and weak (calculated from Py-IR spectra recorded at 350 °C, within 250–350 °C, and within 150–250 °C, respectively).

## References

[1] Díaz U. (2012). Layered Materials with Catalytic Applications Pillared and Delaminated Zeolites. Int. Sch. Res. Netw..

[2] Yang W., Wang Z., Sun H., Zhang B. (2016). Advances in Development and Industrial Applications of Ethylbenzene Processes. Cuihua Xuebao Chin. J. Catal..

[3] Xu L., Sun J. (2016). Recent Advances in the Synthesis and Application of Two-Dimensional Zeolites. Adv. Energy Mater..

[4] Riaz F., Guarino C.H.L. (1999). Mobil/Badger Cumene Process. Hydrocarb. Eng..

[5] Rutkowska M., Díaz U., Palomares A.E., Chmielarz L. (2015). Cu and Fe Modified Derivatives of 2D MWW-Type Zeolites (MCM-22, ITQ-2 and MCM-36) as New Catalysts for DeNOx Process. Appl. Catal. B Environ..

[6] Bennett J.M., Chang C.D., Lawton S.L., Leonowicz M.E., Lissy D.N., Rubin M.K. (1993). Synthetic Porous Crystalline MCM-49, Its Synthesis and Use.

[7] Ostroumova V.A., Maksimov A.L. (2019). MWW-Type Zeolites: MCM-22, MCM-36, MCM-49, and MCM-56 (A Review). Pet. Chem..

[8] Corma A., Fornés V., Guil J.M., Pergher S., Maesen T.L.M., Buglass J.G. (2000). Preparation, Characterisation and Catalytic Activity of ITQ-2, a Delaminated Zeolite. Microporous Mesoporous Mater..

[9] Szymaszek-Wawryca A., Díaz U., Samojeden B., Motak M. (2022). Catalytic Performance of One-Pot Synthesized Fe-MWW Layered Zeolites (MCM-22, MCM-36, and ITQ-2) in Selective Catalytic Reduction of Nitrogen Oxides with Ammonia. Molecules.

[10] Marosz M., Samojeden B., Kowalczyk A., Rutkowska M., Motak M., Díaz U., Palomares A.E., Chmielarz L. (2020). MCM-22, MCM-36, and ITQ-2 Zeolites with Different Si/Al Molar Ratios as Effective Catalysts of Methanol and Ethanol Dehydration. Materials.

[11] Kikhtyanin O., Chlubná P., Jindrova T., Kubicka D. (2014). Peculiar Behavior of MWW Materials Aldol Condensation of Furfural and Acetone—MCM-22 MCM-36. Dalton Trans..

[12] Kaskow I., Wojtaszek-gurdak A., Sobczak I. (2020). Methanol Oxidation on AuAg-Zn/MCM-36—The Effect of Catalyst Components and Pretreatment. Catal. Today.

[13] Jankowska A., Kowalczyk A., Rutkowska M., Mozgawa W., Gil B., Chmielarz L. (2020). Silica and Silica-Titania Intercalated MCM-36 Modified with Iron as Catalysts for Selective Reduction of Nitrogen Oxides-The Role of Associated Reactions. Catal. Sci. Technol..

[14] Kresge C.T., Roth W.J., Simmons K.G., Vartuli J.C. (1993). Crystalline Oxide Material. US Patent.

[15] Zhang Z., Zhu W., Zai S., Jia M., Zhang W., Wang Z. (2013). Synthesis, Characterization and Catalytic Properties of MCM-36 Pillared via the MCM-56 Precursor. J. Porous Mater..

[16] Ahmad N., Hussain S.T., Muhammad B., Ali N., Abbas S.M., Ali Z. (2013). Zr-Pillared Montmorillonite Supported Cobalt Nanoparticles for Fischer–Tropsch Synthesis. Prog. Nat. Sci. Mater. Int..

[17] Chen Q., Wu P., Dang Z., Zhu N., Li P., Wu J., Wang X. (2010). Iron Pillared Vermiculite as a Heterogeneous Photo-Fenton Catalyst for Photocatalytic Degradation of Azo Dye Reactive Brilliant Orange X-GN. Sep. Purif. Technol..

[18] Kornatowski J., Barth J.O., Erdmann K., Rozwadowski M. (2006). Effect of Various Pillaring Oxides on Adsorption Behaviour of Novel MCM-36 Derivatives. Microporous Mesoporous Mater..

[19] Barth J., Kornatowski J., Lercher J.A. (2002). Synthesis of New MCM-36 Derivatives Pillared with Alumina or Magnesia—Alumina. J. Mater. Chem..

[20] Barth J., Jentys A., Kornatowski J., Lercher J.A., Al O., Omar M. (2004). Al Control of Acid—Base Properties of New Nanocomposite Derivatives of MCM-36 by Mixed Oxide Pillaring. Chem. Mater..

[21] Wang T., Jin F., Yi X., Wu G., Zheng A. (2021). Atom-Planting Synthesis of MCM-36 Catalyst to Investigate the Influence of Pore Structure and Titanium Coordination State on Epoxidation Activity. Microporous Mesoporous Mater..

[22] Jin F., Chen S.Y., Jang L.Y., Lee J.F., Cheng S. (2014). New Ti-Incorporated MCM-36 as an Efficient Epoxidation Catalyst Prepared by Pillaring MCM-22 Layers with Titanosilicate. J. Catal..

[23] Jin F., Huang S., Cheng S., Wu Y., Chang C.C., Huang Y.W. (2015). The Influences of Al Species and Ti Species on the Catalytic Epoxidation over Si/Ti-Pillared MCM-36 Synthesized from MCM-22. Catal. Sci. Technol..

[24] Szymaszek A., Samojeden B., Motak M. (2020). The Deactivation of Industrial SCR Catalysts—A Short Review. Energies.

[25] Zyrkowski M., Motak M., Samojeden B., Szczepanek K. (2020). Deactivation of V2O5-WO3/TiO2 DeNOx Catalyst under Commercial Conditions in Power Production Plant. Energies.

[26] Liang J., Hu W.F., Song B., Mou T., Zhang L., Luo Y., Liu Q., Alshehri A.A., Hamdy M.S., Yang L.M. (2022). Efficient Nitric Oxide Electroreduction toward Ambient Ammonia Synthesis Catalyzed by a CoP Nanoarray. Inorg. Chem. Front..

[27] Liang J., Chen H., Mou T., Zhang L., Lin Y., Yue L., Luo Y., Liu Q., Li N., Alshehri A.A. (2022). Coupling Denitrification and Ammonia Synthesis via Selective Electrochemical Reduction of Nitric Oxide over Fe2O3 Nanorods. J. Mater. Chem. A Mater..

[28] Zhang L., Liang J., Wang Y., Mou T., Lin Y., Yue L., Li T., Liu Q., Luo Y., Li N. (2021). High-Performance Electrochemical NO Reduction into NH3 by MoS2 Nanosheet. Angew. Chem.—Int. Ed..

[29] Szymaszek-Wawryca A., Diaz U., Duraczyńska D., Świerczek K., Samojeden B., Motak M. (2022). Catalytic Performance and Sulfur Dioxide Resistance of One-Pot Synthesized Fe-MCM-22 in Selective Catalytic Reduction of Nitrogen Oxides with Ammonia (NH3-SCR)—The Effect of Iron Content. Int. J. Mol. Sci..

[30] Li K., Lei J., Yuan G., Weerachanchai P., Wang J.Y., Zhao J., Yang Y. (2017). Fe-, Ti-, Zr- and Al-Pillared Clays for Efficient Catalytic Pyrolysis of Mixed Plastics. Chem. Eng. J..

[31] Kizilduman B.K., Alkan M., Doğan M., Turhan Y. (2017). Al-Pillared-Montmorillonite (AlPMt)/Poly(Methyl Methacrylate)(PMMA) Nanocomposites: The Effects of Solvent Types and Synthesis Methods. Adv. Mater. Sci..

[32] Banković P., Milutinović-Nikolić A., Mojović Z., Jović-Jovičić N., Žunić M., Dondur V., Jovanović D. (2012). Al,Fe-Pillared Clays in Catalytic Decolorization of Aqueous Tartrazine Solutions. Appl. Clay Sci..

[33] Barrault J., Abdellaoui M., Bouchoule C., Majesté A., Tatibouët J.M., Louloudi A., Papayannakos N., Gangas N.H. (2000). Catalytic Wet Peroxide Oxidation over Mixed (Al-Fe) Pillared Clays. Appl. Catal. B Environ..

[34] Boroń P., Chmielarz L., Gurgul J., Łątka K., Gil B., Marszałek B., Dzwigaj S. (2015). Influence of Iron State and Acidity of Zeolites on the Catalytic Activity of FeHBEA, FeHZSM-5 and FeHMOR in SCR of NO with NH_3_ and N_2_O Decomposition. Microporous Mesoporous Mater..

[35] Chen J., Peng G., Zheng W., Zhang W., Guo L., Wu X. (2020). Excellent Performance of One-Pot Synthesized Fe-Containing MCM-22 Zeolites for the Selective Catalytic Reduction of NO: Xwith NH3. Catal. Sci. Technol..

[36] Mauricio B., Andrade H.M.C., Mascarenhas A.J.S. (2019). Oxidative Dehydration of Glycerol over Alternative H,Fe-MCM-22 Catalysts: Sustainable Production of Acrylic Acid. Microporous Mesoporous Mater..

[37] Roth W.J., Gil B., Makowski W., Sławek A., Korzeniowska A., Grzybek J., Siwek M., Michorczyk P. (2016). Framework-Substituted Cerium MCM-22 Zeolite and Its Interlayer Expanded Derivative MWW-IEZ. Catal. Sci. Technol..

[38] Chmielarz L., Kuśtrowski P., Piwowarska Z., Michalik M., Dudek B., Dziembaj R. (2009). Natural Micas Intercalated with Al2O3 and Modified with Transition Metals as Catalysts of the Selective Oxidation of Ammonia to Nitrogen. Top. Catal..

[39] Kim S.H., Komarneni S., Heo N.H. (2011). ZSM-5 and Ferrierite Single Crystals with Lower Si/Al Ratios: Synthesis and Single-Crystal Synchrotron X-Ray Diffraction Studies. Microporous Mesoporous Mater..

[40] Thommes M., Kaneko K., Neimark A.V., Olivier J.P., Rodriguez-Reinoso F., Rouquerol J., Sing K.S.W. (2015). Physisorption of Gases, with Special Reference to the Evaluation of Surface Area and Pore Size Distribution (IUPAC Technical Report). Pure Appl. Chem..

[41] He Y.J., Nivarthy G.S., Eder F., Seshan K., Lercher J.A. (1998). Synthesis, Characterization and Catalytic Activity of the Pillared Molecular Sieve MCM-36. Microporous Mesoporous Mater..

[42] Chlubná P., Roth W.J., Zukal A., Kubu M., Jules J.P. (2012). Pillared MWW Zeolites MCM-36 Prepared by Swelling MCM-22P in Concentrated Surfactant Solitions. Catal. Today.

[43] Maheshwari S., Jordan E., Kumar S., Bates F.S., Penn R.L., Shantz D.F., Tsapatsis M. (2008). Layer Structure Preservation during Swelling, Pillaring, and Exfoliation of a Zeolite Precursor. J. Am. Chem. Soc..

[44] Thakkar R., Bandyopadhyay R. (2017). Preparation, Characterization, and Post-Synthetic Modification of Layered MCM-22 Zeolite Precursor. J. Chem. Sci..

[45] Sobhanardakani S., Jafari A., Zandipak R., Meidanchi A. (2018). Removal of Heavy Metal (Hg(II) and Cr(VI)) Ions from Aqueous Solutions Using Fe_2_O_3_@SiO_2_ Thin Films as a Novel Adsorbent. Process. Saf. Environ. Prot..

[46] Carriço C.S., Cruz F.T., Santos M.B., Pastore H.O., Andrade H.M.C., Mascarenhas A.J.S. (2013). Efficiency of Zeolite MCM-22 with Different SiO_2_/Al_2_O_3_ Molar Ratios in Gas Phase Glycerol Dehydration to Acrolein. Microporous Mesoporous Mater..

[47] Juybar M., Khanmohammadi Khorrami M., Bagheri Garmarudi A., Zandbaaf S. (2020). Determination of Acidity in Metal Incorporated Zeolites by Infrared Spectrometry Using Artificial Neural Network as Chemometric Approach. Spectrochim. Acta—Part A Mol. Biomol. Spectrosc..

[48] Kumar A., Lingfa P. (2020). Sodium Bentonite and Kaolin Clays: Comparative Study on Their FT-IR, XRF, and XRD. Mater. Today Proc..

[49] Onida B., Geobaldo F., Testa F., Aiello R., Garrone E. (2002). H-Bond Formation and Proton Transfer in H-MCM-22 Zeolite as Compared to H-ZSM-5 and H-MOR: An FTIR Study. J. Phys. Chem. B.

[50] Onida B., Geobaldo F., Testa F., Crea F., Garrone E. (1999). FTIR Investigation of the Interaction at 77 K of Diatomic Molecular Probes on MCM-22 Zeolite. Microporous Mesoporous Mater..

[51] Chmielarz L., Kuśtrowski P., Dziembaj R., Cool P., Vansant E.F. (2010). SBA-15 Mesoporous Silica Modified with Metal Oxides by MDD Method in the Role of DeNOx Catalysts. Microporous Mesoporous Mater..

[52] Nawab M., Barot S., Bandyopadhyay R. (2019). Solvent-Free Selective Oxidation of Toluene over Metal-Doped MCM-22. New J. Chem..

[53] Gil B., Marszałek B., Micek-Ilnicka A., Olejniczak Z. (2010). The Influence of Si/Al Ratio on the Distribution of OH Groups in Zeolites with MWW Topology. Top. Catal..

[54] Mihályi R.M., Lázár K., Kollár M., Lónyi F., Pál-Borbély G., Szegedi Á. (2008). Structure, Acidity and Redox Properties of MCM-22 Ferrisilicate. Microporous Mesoporous Mater..

[55] Corma A., Corell C., Kolodziejski W., Prez-pariente J., Quimica I.D.T., Polit U., Valencia D. (1995). Infrared Spectroscopy, Acidity, Structure, and Stability Of Zeolites. Microporous Mesoporous Mater..

[56] Grijndling C., Gründling L., Eder-Mirth P. (1996). Infrared Studies of the Surface Acidity of Oxides and Zeolites Using Adsorbed Probe Molecules.Pdf. Catal. Today.

[57] Góra-Marek K., Datka J. (2006). IR Studies of OH Groups in Mesoporous Aluminosilicates. Appl. Catal. A Gen..

[58] Usman M., Li D., Li C., Zhang S. (2015). Highly Selective and Stable Hydrogenation of Heavy Aromatic-Naphthalene over Transition Metal Phosphides. Sci. China Chem..

[59] Peng Y., Wang C., Li J. (2014). Structure-Activity Relationship of VOx/CeO2 Nanorod for NO Removal with Ammonia. Appl. Catal. B Environ..

[60] Chen J., Liang T., Li J., Wang S., Qin Z., Wang P., Huang L., Fan W., Wang J. (2016). Regulation of Framework Aluminum Siting and Acid Distribution in H–MCM-22 by Boron Incorporation and Its Effect on the Catalytic Performance in Methanol to Hydrocarbons. ACS Catal..

[61] Jenness G.R., Christiansen M.A., Caratzoulas S., Vlachos D.G., Gorte R.J. (2014). Site-Dependent Lewis Acidity of γ-Al2O3 and Its Impact on Ethanol Dehydration and Etherification. J. Phys. Chem. C.

[62] Stephenson N.A., Bell A.T. (2007). Mechanistic Insights into Iron Porphyrin-Catalyzed Olefin Epoxidation by Hydrogen Peroxide: Factors Controlling Activity and Selectivity. J. Mol. Catal. A Chem..

[63] Hu Q., Huang X., Cui Y., Luo T., Tang X., Wang T., Qian W., Wei F. (2017). High Yield Production of C2-C3 Olefins and: Para- Xylene from Methanol Using a SiO_2_-Coated FeOx/ZSM-5 Catalyst. RSC Adv..

[64] Guisnet M., De Poitiers U., Pineau R., Juin M. (1974). Chapter 1. Introduction to Zeolite Science and Technology. Zeolites for Cleaner Technologies.

[65] Palčić A., Valtchev V. (2020). Analysis and Control of Acid Sites in Zeolites. Appl. Catal. A Gen..

[66] Macina D., Piwowarska Z., Góra-Marek K., Tarach K., Rutkowska M., Girman V., Błachowski A., Chmielarz L. (2016). SBA-15 Loaded with Iron by Various Methods as Catalyst for DeNOx Process. Mater. Res. Bull..

[67] Rutkowska M., Jankowska A., Różycka-Dudek E., Dubiel W., Kowalczyk A., Piwowarska Z., Llopis S., Díaz U., Chmielarz L. (2020). Modification of Mcm-22 Zeolite and Its Derivatives with Iron for the Application in N2o Decomposition. Catalysts.

[68] Liu Q., Bian C., Ming S., Guo L., Zhang S., Pang L., Liu P., Chen Z., Li T. (2020). The Opportunities and Challenges of Iron-Zeolite as NH3-SCR Catalyst in Purification of Vehicle Exhaust. Appl. Catal. A Gen..

[69] Ryu T., Hong S.B. (2020). Iron-Exchanged UZM-35: An Active NH3-SCR Catalyst at Low Temperatures. Appl. Catal. B Environ..

[70] Yang S., Liu C., Chang H., Ma L., Qu Z., Yan N., Wang C., Li J. (2013). Improvement of the Activity of γ-Fe_2_O_3_ for the Selective Catalytic Reduction of NO with NH_3_ at High Temperatures: NO Reduction versus NH_3_ Oxidization. Ind. Eng. Chem. Res..

[71] Chmielarz L., Kowalczyk A., Wojciechowska M., Boroń P., Dudek B., Michalik M. (2014). Montmorillonite Intercalated with SiO_2_, SiO_2_-Al_2_O_3_ or SiO_2_-TiO_2_ Pillars by Surfactant-Directed Method as Catalytic Supports for DeNOx Process. Chem. Pap..

[72] Brandenberger S., Kröcher O., Tissler A., Althoff R. (2010). The Determination of the Activities of Different Iron Species in Fe-ZSM-5 for SCR of NO by NH_3_. Appl. Catal. B Environ..

[73] Busca G., Lietti L., Ramis G., Berti F. (1998). Chemical and Mechanistic Aspects of the Selective Catalytic Reduction of NO(x) by Ammonia over Oxide Catalysts: A Review. Appl. Catal. B Environ..

[74] Koebel M., Madia G., Elsener M. (2002). Selective Catalytic Reduction of NO and N_2_O at Low Temperatures. Catal. Today.

[75] Delahay G., Mauvezin M., Coq B., Kieger S. (2001). Selective Catalytic Reduction of Nitrous Oxide by Ammonia on Iron Zeolite Beta Catalysts in an Oxygen Rich Atmosphere: Effect of Iron Contents. J. Catal..

[76] Ben Younes N., Ortigosa J.M., Marie O., Blasco T., Mhamdi M. (2021). Effect of Zeolite Structure on the Selective Catalytic Reduction of NO with Ammonia over Mn-Fe Supported on ZSM-5, BEA, MOR and FER. Res. Chem. Intermed..

[77] Giakoumelou I., Fountzoula C., Kordulis C., Boghosian S. (2006). Molecular Structure and Catalytic Activity of V_2_O_5_/TiO_2_ Catalysts for the SCR of NO by NH_3_: In Situ Raman Spectra in the Presence of O_2_, NH_3_, NO, H_2_, H_2_O, and SO_2_. J. Catal..

[78] Topsoe N.-Y., Dumesic J.A., Topsøe H. (1995). Vanadia/Titania Catalysts for Selective Catalytic Reduction of NItric Oxide by Ammonia II. Stud. Act. Sites Formul. Catal. Cycles.

[79] Mou X., Zhang B., Li Y., Yao L., Wei X., Su D.S., Shen W. (2012). Rod-Shaped Fe_2_O_3_ as an Efficient Catalyst for the Selective Reduction of Nitrogen Oxide by Ammonia. Angew. Chem.—Int. Ed..

[80] Wang Y., Lei Z., Chen B., Guo Q., Liu N. (2010). Adsorption of NO and N 2 O on Fe-BEA and H-BEA Zeolites. Appl. Surf. Sci..

[81] Long R.Q., Yang R.T. (2000). Characterization of Fe-ZSM-5 Catalyst for Selective Catalytic Reduction of Nitric Oxide by Ammonia. J. Catal..

[82] Schwidder M., Santhosh Kumar M., Bentrup U., Pérez-Ramírez J., Brückner A., Grünert W. (2008). The Role of Brønsted Acidity in the SCR of NO over Fe-MFI Catalysts. Microporous Mesoporous Mater..

[83] Amores J.M.G., Sanchez Escribano V., Ramis G., Busca G. (1997). An FT-IR Study of Ammonia Adsorption and Oxidation over Anatase-Supported Metal Oxides. Appl. Catal. B Environ..

[84] Brandenberger S., Kröcher O., Wokaun A., Tissler A., Althoff R. (2009). The Role of Brønsted Acidity in the Selective Catalytic Reduction of NO with Ammonia over Fe-ZSM-5. J. Catal..

[85] Liu Z., Millington P.J., Bailie J.E., Rajaram R.R., Anderson J.A. (2007). A Comparative Study of the Role of the Support on the Behaviour of Iron Based Ammonia SCR Catalysts. Microporous Mesoporous Mater..

[86] Qu W., Chen Y., Huang Z., Gao J., Zhou M., Chen J., Li C., Ma Z., Chen J., Tang X. (2017). Active Tetrahedral Iron Sites of γ-Fe2O3 Catalyzing NO Reduction by NH3. Environ. Sci. Technol. Lett..

[87] Liu F., He H., Zhang C., Shan W., Shi X. (2011). Mechanism of the Selective Catalytic Reduction of NOx with NH3 over Environmental-Friendly Iron Titanate Catalyst. Catal. Today.

[88] Apostolescu N., Geiger B., Hizbullah K., Jan M.T., Kureti S., Reichert D., Schott F., Weisweiler W. (2006). Selective Catalytic Reduction of Nitrogen Oxides by Ammonia on Iron Oxide Catalysts. Appl. Catal. B Environ..

[89] Zhang D., Yang R.T. (2018). N2O Formation Pathways over Zeolite-Supported Cu and Fe Catalysts in NH3-SCR. Energy Fuels.

[90] Corma A., Corell C. (1995). Synthesis and Characterization of the MCM-22 Zeolite. Zeolites.

[91] Muñoz H.J., Blanco C., Gil A., Vicente M.Á., Galeano L.A. (2017). Preparation of Al/Fe-Pillared Clays: Effect of the Starting Mineral. Materials.

[92] Rouquerol J., Llewellyn P., Rouquerol F. (2007). Is the BET Equation Applicable to Microporous Adsorbents?. Stud. Surf. Sci. Catal..

[93] Emeis C.A. (1993). Determination of Integrated Molar Extinction Coefficients for Infrared Absorption Bands of Pyridine Adsorbed on Solid Acid Catalysts. J. Catal..

