# Supplementary material for: Synthesis, Characterization, and NH3-SCR Catalytic Performance of Fe-Modified MCM-36 Intercalated with Various Pillars
Source: Molecules. 2023 Jun 24;28(13):4960. doi: 10.3390/molecules28134960 (PMC10343675; doi:10.3390/molecules28134960)
Supplement: Supplementary file 1 [file molecules-28-04960-s001.zip › Supplementary_Molecules.pdf]

Synthesis, characterization, and NH_3-SCR catalytic performance of Fe-modified MCM-36 intercalated with various pillars

Agnieszka Szymaszek-Wawryca [1,*], Urbano Díaz [2], Bogdan Samojeden[1], and Monika Motak [1]

[1] *AGH University of Science and Technology, Faculty of Energy and Fuels, al. Mickiewicza 30, 30-059 Kraków, Poland*

[2] *Instituto de Tecnología Química, UPV-CSIC, Universidad Politécnica de Valencia, Avenida de los Naranjos, s/n. 46022 Valencia, Spain*

* corresponding author: agnszym@agh.edu.pl

Supplementary material

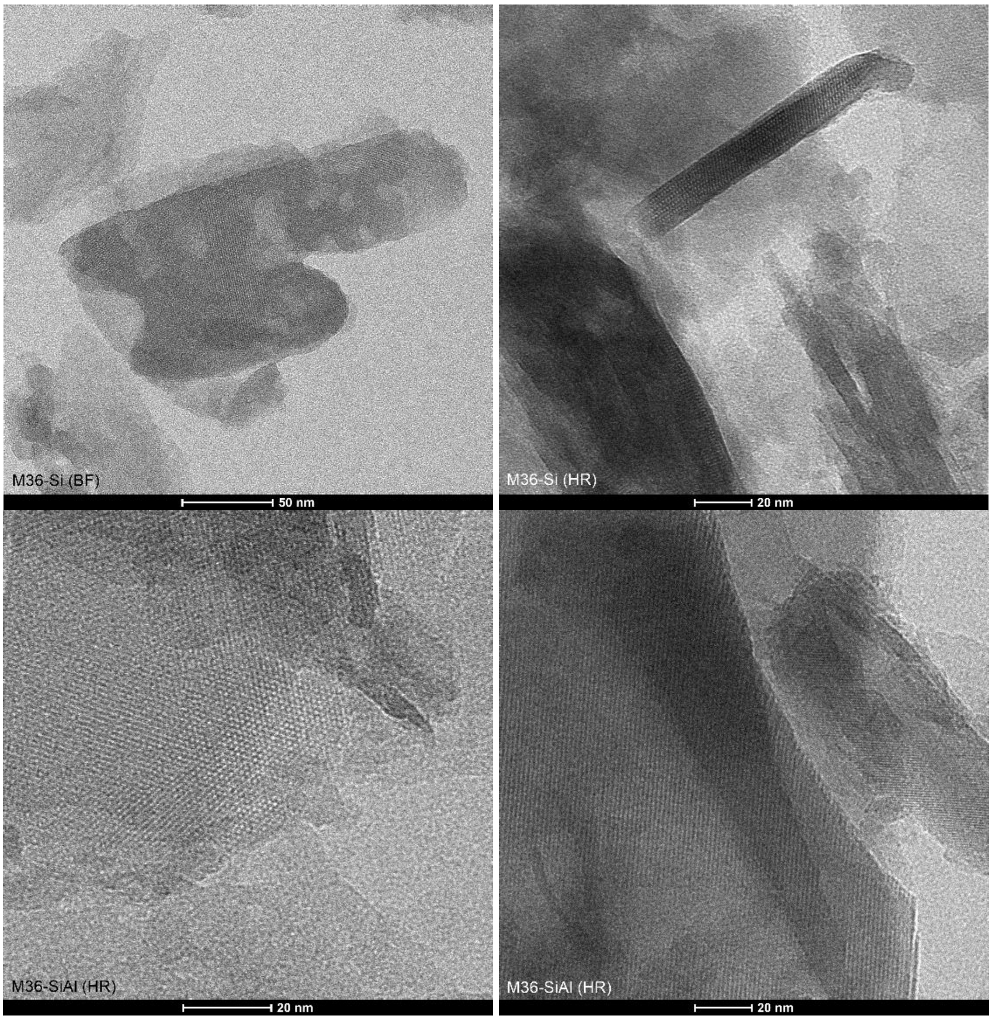

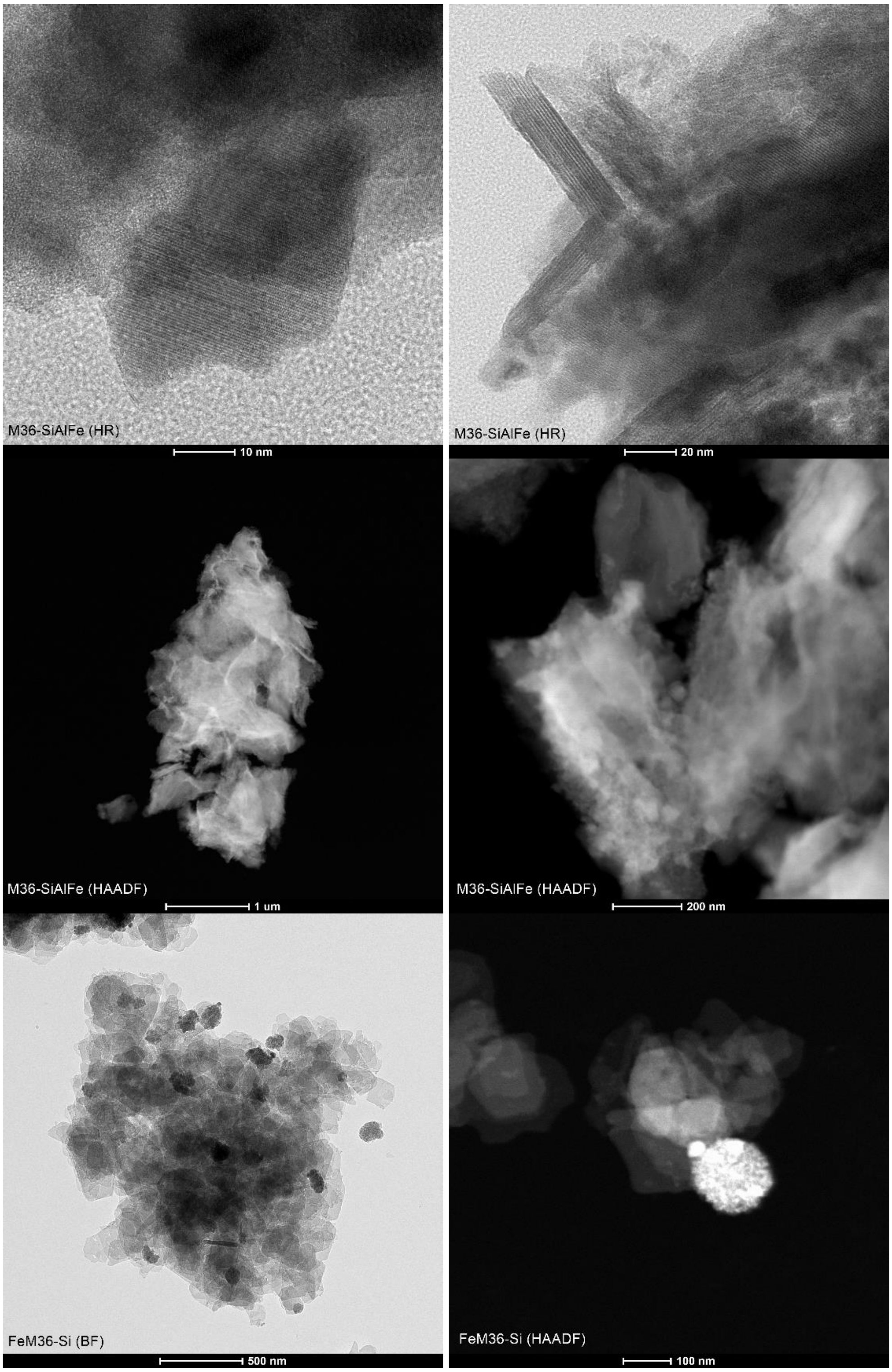
M36-SiAlFe (HR)
10 nm
M36-SiAlFe (HR)
20 nm
M36-SiAlFe (HAADF)
1 um
M36-SiAlFe (HAADF)
200 nm
FeM36-Si (BF)
500 nm
FeM36-Si (HAADF)
100 nm

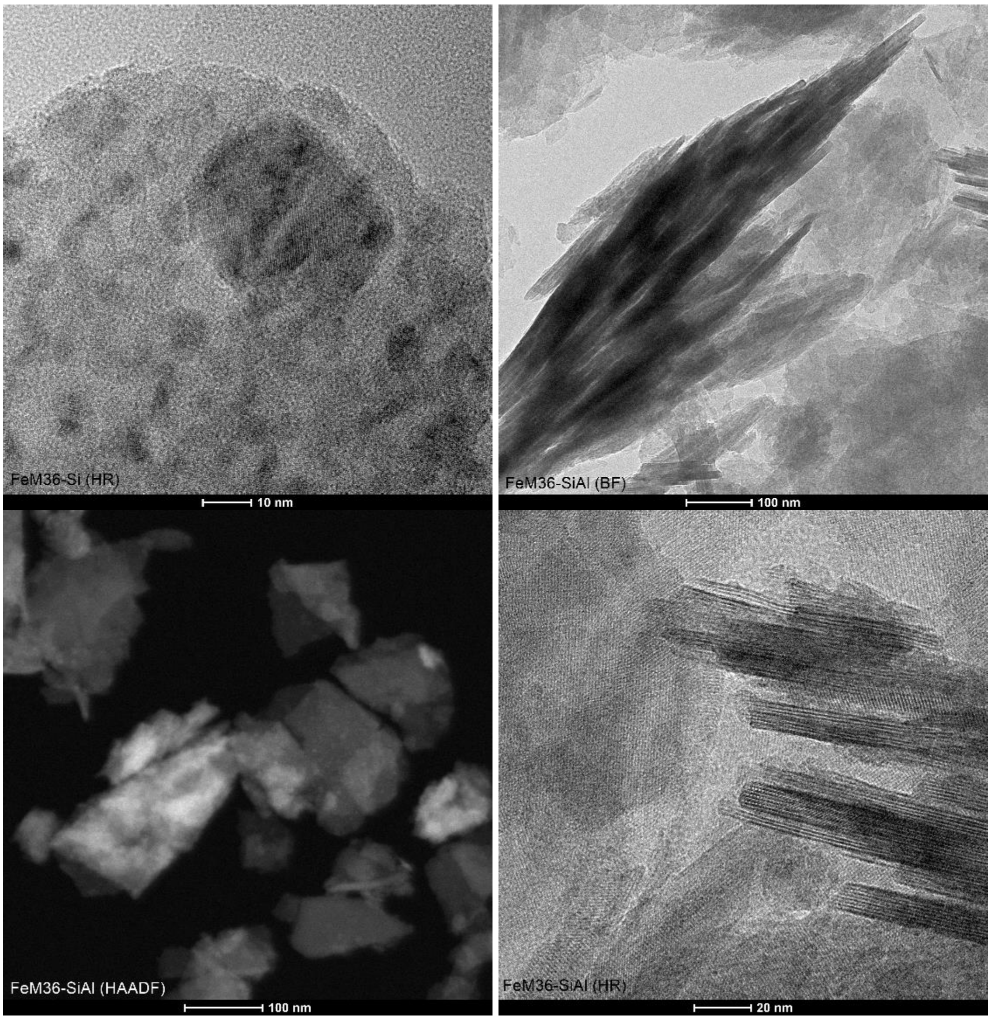

Figure S1. TEM images of the samples recorded in various modes.

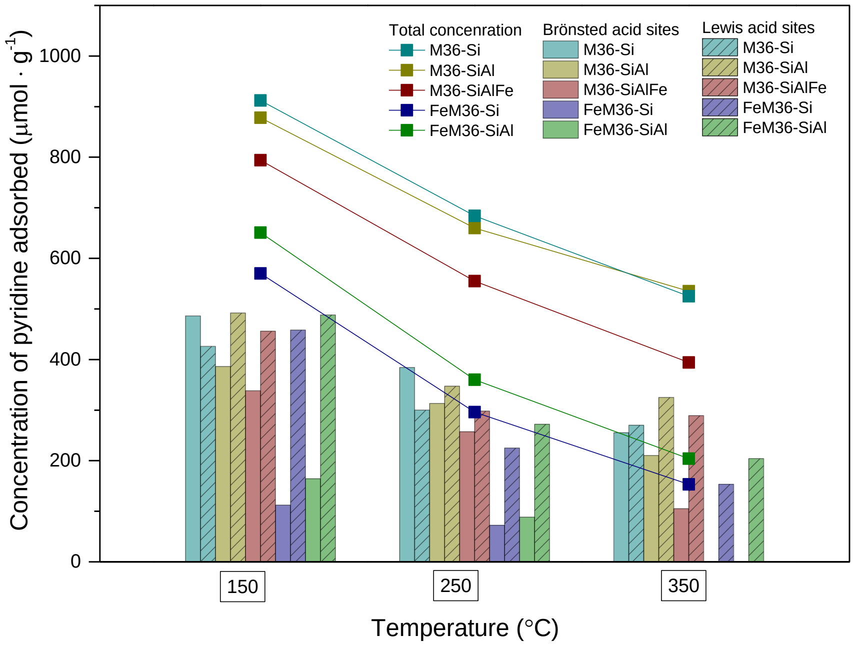

Figure S2. Changes in the concentration of pyridine adsorbed on the acid centers of the materials at 150, 250, and 350 °C.

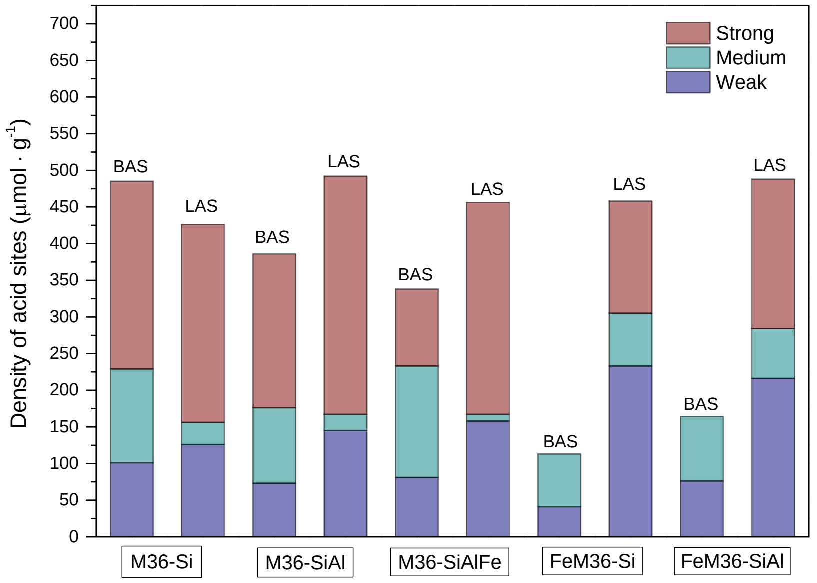

Figure S3. Densities of Brönsted (BAS) and Lewis (LAS) acid sites in relation to their strength: strong, medium, weak (calculated from Py-IR spectra recorded at 350 °C, within 250-350 °C, and within 150-250 °C, respectively).
